# Fgf and Esrrb integrate epigenetic and transcriptional networks that regulate self-renewal of trophoblast stem cells

**DOI:** 10.1038/ncomms8776

**Published:** 2015-07-24

**Authors:** Paulina A. Latos, Angela Goncalves, David Oxley, Hisham Mohammed, Ernest Turro, Myriam Hemberger

**Affiliations:** 1 Epigenetics Programme, The Babraham Institute, Babraham Research Campus, Cambridge CB22 3AT, UK; 2 Centre for Trophoblast Research, University of Cambridge, Downing Street, Cambridge CB2 3EG, UK; 3 Wellcome Trust Sanger Institute, Cambridge CB10 1SA, UK; 4 Proteomics Group, The Babraham Institute, Babraham Research Campus, Cambridge CB22 3AT, UK; 5 Department of Haematology, University of Cambridge, NHS Blood and Transplant, Long Road, Cambridge CB2 0PT, UK; 6 Medical Research Council Biostatistics Unit, Cambridge Institute of Public Health, Robinson Way, Forvie Site, Cambridge CB2 0SR, UK

## Abstract

Esrrb (oestrogen-related receptor beta) is a transcription factor implicated in embryonic stem (ES) cell self-renewal, yet its knockout causes intrauterine lethality due to defects in trophoblast development. Here we show that in trophoblast stem (TS) cells, *Esrrb* is a downstream target of fibroblast growth factor (Fgf) signalling and is critical to drive TS cell self-renewal. In contrast to its occupancy of pluripotency-associated loci in ES cells, Esrrb sustains the stemness of TS cells by direct binding and regulation of TS cell-specific transcription factors including *Elf5* and *Eomes*. To elucidate the mechanisms whereby Esrrb controls the expression of its targets, we characterized its TS cell-specific interactome using mass spectrometry. Unlike in ES cells, Esrrb interacts in TS cells with the histone demethylase Lsd1 and with the RNA Polymerase II-associated Integrator complex. Our findings provide new insights into both the general and context-dependent wiring of transcription factor networks in stem cells by master transcription factors.

## Abstract (web summary)

The transcription factor estrogen-related receptor beta, Esrrb, regulates pluripotency genes in embryonic stem cells, but how it acts in trophoblast stem (TS) cells is unclear. Here, the authors identify Esrrb as a primary target of Fgf/Mek signaling and outline a unique TS cell-specific interactome to sustain stemness.

## Graphical abstract

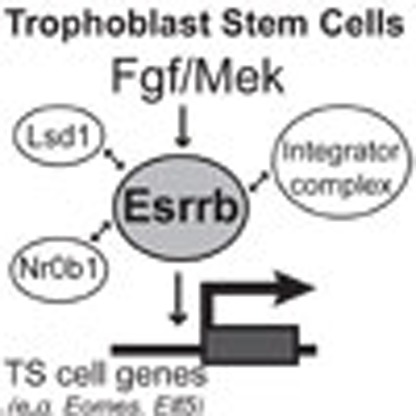

The placenta is an essential organ that ensures the exchange of nutrients, oxygen, hormones, metabolic by-products and other molecules between the maternal and fetal bloodstreams^1^. Essential insights into the molecular pathways controlling placental development have been gained by using trophoblast stem (TS) cells that can self-renew and differentiate into the various placental trophoblast cell types *in vitro*^2^,^3^. Mouse TS cells are derived from the trophectoderm of blastocysts and represent the developmental counterpart to embryonic stem (ES) cells derived from the preimplantation epiblast. Unlike ES cells, TS cells can also be derived from the extraembryonic ectoderm of early post-implantation conceptuses^2^,^4^. Derivation and maintenance of TS cells depends on fibroblast growth factor (Fgf) and Nodal/Activin signalling^2^,^5^,^6^,^7^. Consequently, the withdrawal of both components leads to the differentiation of TS cells into various trophoblast cell types of the chorioallantoic placenta including spongiotrophoblast, syncytiotrophoblast and giant cells^2^.

In TS cells, Fgf signalling predominantly stimulates the Mek/Erk pathway leading to the expression of essential TS cell-specific transcription factors (TFs) such as Cdx2 (refs 2, 8, 9). In addition to Cdx2, other key TFs that are critical to maintain the stem cell state of TS cells include Eomes, Esrrb, Elf5, Sox2 and Tfap2c (refs 10, 11, 12, 13, 14, 15). Interestingly, some of these, such as Eomes, Elf5 and Tfap2c, have seemingly TS cell-specific functions during this developmental window, whereas others, notably Sox2 and Esrrb, have pivotal roles also in regulating pluripotency of ES cells^11^,^12^,^13^,^14^,^15^,^16^,^17^.

Recent findings suggest that the requirement for Fgf (Fgf4) signalling in TS cells cannot be replaced by the ectopic expression of a single one of these TFs (that is, Elf5, Eomes, Cdx2, Tfap2c, Sox2 or Esrrb). However, the combined ectopic expression of Sox2 and Esrrb has been shown to be capable of sustaining TS cell self-renewal in the absence of Fgf4 (ref. 18). While Sox2 functions by interacting with Tfap2c, which in turn recruits Sox2 to Fgf-regulated genes, the critical interactors of Esrrb in TS cells remain unknown^18^.

Esrrb (oestrogen-related receptor beta) plays a key role in trophoblast development as embryos deficient for *Esrrb* die before E10.5 because of severely impaired placental formation, characterized by an abnormal chorion layer and overabundance of giant cells^12^. In line with a pivotal role in trophoblast development, TS cells cannot be derived from *Esrrb* mutants^19^. Tetraploid aggregation experiments proved that the embryonic lethality can be rescued by wild-type (wt) trophoblast cells, thus demonstrating that the essential function of Esrrb during early development resides in the trophoblast compartment.

Although *Esrrb* is dispensable for development of the embryo proper, it is required for self-renewal of mouse ES cells in ground-state conditions^16^,^20^,^21^. In this context, Esrrb cooperates with a range of TFs (e.g., Oct4, Sall4 and Ncoa3), chromatin-remodelling complexes and with components of the transcriptional machinery including the Mediator complex and RNA Polymerase II (RNAPII) to regulate self-renewal^20^,^22^,^23^. Thus, similar to *Sox2*, *Esrrb* is a key TF in both ES and TS cells, raising questions about its specificity in different developmental contexts and whether it acts as a more general determinant of stemness irrespective of stem cell type.

Here we address the function of *Esrrb* in TS cells. We show that the regulation and target gene network differ profoundly between ES and TS cells. Unlike in ES cells, *Esrrb* is the most prominent early-response gene to Mek inhibition in TS cells, the main downstream effector of Fgf signalling in the trophoblast compartment. We demonstrate that *Esrrb* depletion results in downregulation of the key TS cell-specific TFs, consequently causing TS cell differentiation. This function of Esrrb is exerted by directly binding, and activating, a core set of TS cell-specific target genes including *Elf5*, *Eomes*, *Bmp4* and *Sox2*, with little overlap to its chromatin occupancy in ES cells. Finally, by characterizing the Esrrb protein interactome we discovered a number of novel, TS cell-specific interactions. Unlike in ES cells, Esrrb interacts in TS cells with the histone demethylase Lsd1 and with the RNAPII-associated Integrator complex. Taken together, our data reveal that Esrrb regulates highly stem cell-type-specific networks due to distinct interaction partners that are essential to maintain the self-renewal state of TS cells.

## Results

### *Esrrb* is an early target of Fgf/Erk signalling in TS cells

**Figure 1:**
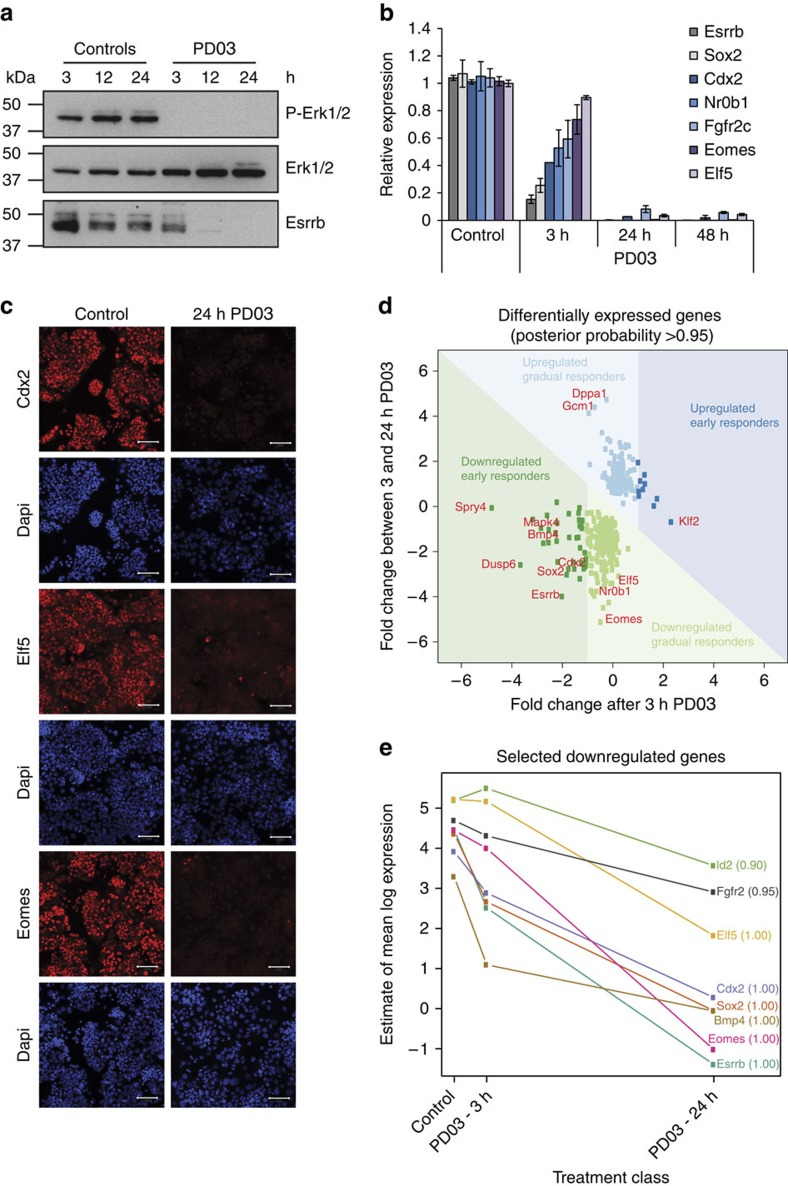
Effects of Fgf/Erk signalling inhibition on TS cell transcription factors. (**a**) Western blot analysis showing the absence of phosphorylated Erk1/2 in cells treated with Mek inhibitor PD0325901 (‘PD03’) for 3, 12 and 24 h compared with untreated controls; levels of total Erk1/2 remained unchanged. Esrrb was reduced after 3 h of PD03 treatment and nearly absent after 12 h of PD03 treatment (Supplementary Fig. 10a). (**b**) RT–QPCR showing expression of TS cell markers in TS cells treated with PD03 for 3, 24 and 48 h compared with untreated controls. *Esrrb* was the most rapidly downregulated gene. Bars represent the mean of three biological replicates±s.e.m. (**c**) Immunostaining of Fgf-responsive transcription factors Cdx2, Elf5 and Eomes in TS cells treated with PD03 for 24 h and untreated controls. Magnification bars, 100 μm. (**d**) RNA-seq analysis after 3 and 24 h of PD03 treatment compared with untreated controls identified a total of 399 deregulated genes at high-confidence (posterior probability score >0.95) that could be grouped into early (significantly changed, using these parameters, after 3 h) and late (after 24 h) responders. Several example genes are indicated. (**e**) Temporal expression dynamics of a number of selected TS cell genes as identified using RNA-seq analysis. Note that *Esrrb* stands out as the most rapidly downregulated TS cell transcription factor also in this genome-wide approach.

Derivation and maintenance of TS cells depend on the presence of Fgf signalling^2^,^24^. Numerous gene knockout experiments identified the mitogen-activated kinase Mek/Erk branch of the Fgf signalling pathway as predominantly active in both TS cells and extraembryonic ectoderm^18^,^25^,^26^,^27^,^28^. Therefore, we first tested changes in expression of key TS cell TFs on Mek/Erk inhibition using the Mek inhibitor PD0325901 (‘PD03’; Fig. 1a). Among the candidate TFs we examined after 3–48 h of treatment, *Esrrb* was the fastest and most profoundly downregulated gene, followed closely by *Sox2*, in line with a recent report^18^ (Fig. 1b). Some TFs implicated in TS cell maintenance including *Eomes*, *Elf5* and *Cdx2* were also downregulated on Mek inhibition albeit at a slower pace, whereas the expression of others such as *Ets2* or *Tfap2c* remained unchanged. These data were confirmed by immunostaining for some of the most prominent TS cell TFs, namely Cdx2, Elf5, Eomes and Tfap2c (Fig. 1c; Supplementary Fig. 1a). To further refine this analysis and to obtain an unbiased genome-wide coverage of the immediate-early-response genes of Mek inhibition in TS cells, we performed RNA sequencing (RNA-seq) analysis after 3 and 24 h of PD03 treatment. This global expression analysis identified in total 399 genes that were deregulated after 3 and 24 h by Fgf signalling (Fig. 1d; Supplementary Data 1). The majority of these genes were induced by Erk activation as 240 of them were downregulated on Mek inhibition, while only 159 genes were upregulated using stringent confidence parameters (Fig. 1d,e; Supplementary Data 1). Functional gene annotation analysis using MouseMine confirmed that affected genes were specifically enriched for extraembryonic (trophoblast) tissue development, as well as for embryonic lethality and transcriptional control in particular for the downregulated genes (Supplementary Fig. 1b,c). Of particular note were the dynamics of downregulation on Mek inhibition; thus, we identified 38 early responders that were downregulated, but only 10 that were upregulated (Fig. 1d). Notably, of the known TS cell TFs, this analysis confirmed *Esrrb* as the earliest, most rapidly silenced gene on PD03 treatment (Fig. 1e). These results provided a comprehensive overview of Fgf-regulated genes in TS cells and identified many potential candidates with a role in trophoblast development.

The finding that *Esrrb* was the most rapidly downregulated gene after 3 h of PD03 exposure suggested that it may be a direct target of Mek/Erk signalling. Next, we asked whether in addition to Fgf either Nodal/Activin or Bmp4 signalling can also regulate *Esrrb* expression in standard TS cell culture conditions. Because levels of *Esrrb* were not affected by either SB431542 (a Nodal/Activin signalling inhibitor) or LDN (a Bmp signalling inhibitor) treatment, we concluded that, unlike Fgf/Mek signalling, Nodal/Activin and Bmp4 signalling did not directly regulate *Esrrb* expression in TS cells (Supplementary Fig. 1d). Notably, the *Esrrb* sensitivity to Fgf pathway inhibition is TS cell-specific, as PD03 treatment of ES cells does not affect *Esrrb* levels^16^. Instead, in ES cells *Esrrb* expression is strongly induced by the Gsk3-beta inhibitor and Wnt agonist CHIR99021 (CH)^16^. To examine whether Gsk3-beta and Wnt signalling are involved in regulation of *Esrrb* in TS cells, we treated them with either CH or the canonical Wnt inhibitor IWR-1. After 72 h of treatment, we found that *Esrrb* levels were unaffected by either of these compounds (Supplementary Fig. 1e). Hence, the regulation of *Esrrb* diverges profoundly in ES and TS cells, as it is mediated by Gsk3-beta and Erk1/2 signalling, respectively. Taken together, these insights prompted us to investigate the specific function of *Esrrb* in TS cells in greater detail.

### Esrrb is pivotal to maintain the TS cell state

**Figure 2:**
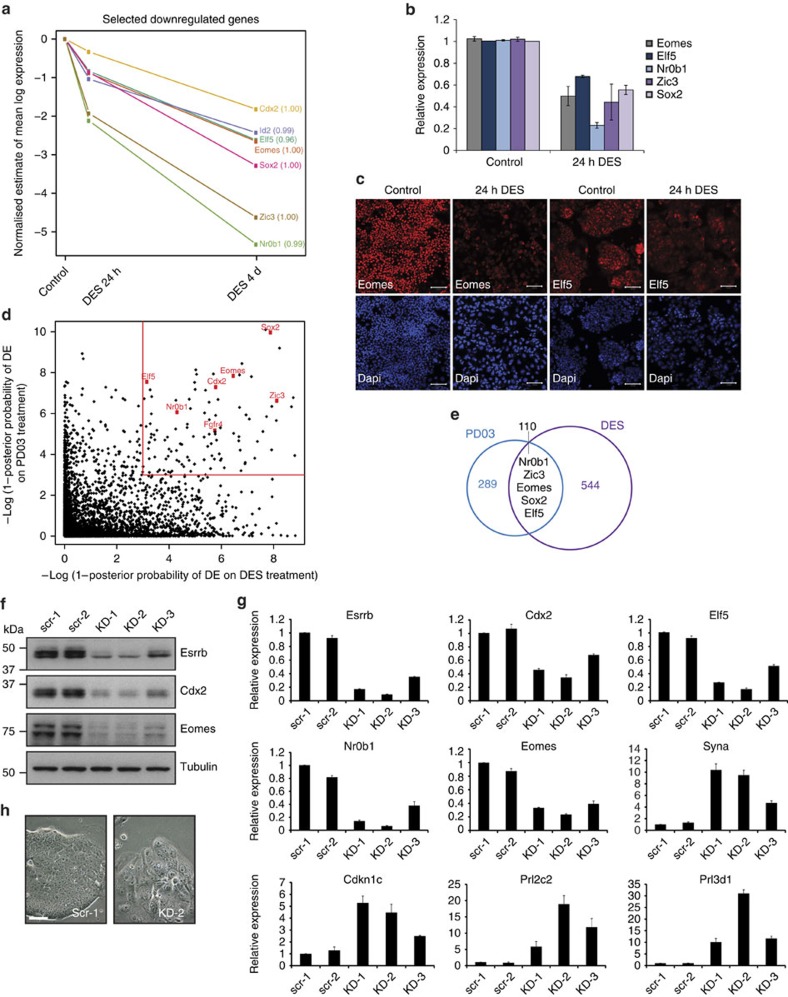
*Esrrb* depletion results in TS cell differentiation. (**a**) Temporal expression dynamics of a number of selected TS cell genes as identified using RNA-seq analysis after 24 h and 4 days of treatment with the oestrogen-related receptor antagonist DES compared with untreated controls. (**b**) RT–QPCR showing expression of TS cell genes in TS cells treated for 24 h with DES compared with untreated controls. (**c**) Immunostaining showing downregulation of TS cell markers Eomes and Elf5 in TS cells treated for 24 h with Esrrb antagonist DES. Magnification bars, 100 μm. (**d**) Plot of differentially expressed genes identified using RNA-seq analysis after 3 and 24 h of PD03 exposure and 4 h and 4-day DES treatment. (**e**) Venn diagram showing overlap of genes deregulated on PD03 and DES treatments. (**f**) Western blot analysis showing depletion of Esrrb, Cdx2 and Eomes in *Esrrb* KD TS cell lines (KD-1, KD-2 and KD-3) compared with controls (scr-1 and scr-2; Supplementary Fig. 10b). (**g**) RT–QPCR analysis of *Esrrb* KD (KD-1, KD-2 and KD-3) and control (scr-1 and scr-2) TS cells. TS cell markers (*Cdx2*, *Elf5*, *Eomes* and *Nr0b1*) were downregulated in *Esrrb*-depleted cells, whereas differentiation markers (*Syna*, *Cdkn1c*, *Prl2c2* and *Prl3d1*) were upregulated. Bars indicate the mean of three biological replicates±s.e.m. (**h**) Phase contrast microscope images of TS cells 5 days after transfection with *Esrrb* KD (KD-2) or scrambled control (scr-1) constructs. *Esrrb* KD lines were severely differentiated despite the presence of Fgf, whereas control lines formed tight, epithelial colonies. These images are representative for KD-1 and KD-2 Esrrb KD lines; KD-3 showed less severe phenotype in line with the reduced KD levels (Fig. 2f,g). Experiments were performed in biological triplicates. Magnification bar, 50 μm.

To gain first insights into which genes may be primary targets of Esrrb, we treated TS cells with the synthetic nonsteroidal oestrogen diethylstilbestrol (DES), an oestrogen-related receptor (Err) antagonist, for 24 h and 4 days. This compound interacts with all three Err isoforms Esrra, Esrrb and Esrrg but mainly acts through Esrrb in early development. It blocks co-activator binding and thus prevents transcriptional activity, and *in vitro* leads to TS cell differentiation^19^. Indeed, we observed morphological changes on DES treatment, indicative of TS cell differentiation. To obtain unbiased genome-wide coverage of transcriptional changes on short (24 h) and prolonged (4d) DES treatment, we performed RNA-seq and identified 654 differentially expressed genes. Numerous differentiation markers were upregulated including the family of placental lactogen genes characteristic for giant cells (Supplementary Data 2). Importantly, we found that transcripts of TS cell TFs *Nr0b1, Zic3, Sox2, Eomes, Elf5* and *Id2* were downregulated after 24 h of DES treatment, suggesting that they may be direct targets of Esrrb (Fig. 2a). We confirmed these findings by reverse transcriptase–quantitative polymerase chain reaction (RT–QPCR) and at the protein level by immunostaining for Eomes and Elf5 (Fig. 2b,c). Interestingly, when specifically examining the trajectories between control and 24 h DES treatment, other prominent TS cell regulators such as *C*dx2 were less influenced during this immediate-response window (Fig. 2a). To further examine Esrrb as a primary mediator of TF induction by Fgf signalling in TS cells, we analysed the overlap of affected genes between the DES and PD03 RNA-seq data sets (Fig. 2d,e). Strikingly, we found that both DES and PD03 treatments had an impact on the same set of prominent stem cell genes *Nr0b1, Zic3, Sox2, Id2*, *Cdx2, Eomes* and *Elf5* (Fig. 2d,e). Taken together, these data indicated that Fgf-Mek signalling regulates, via Esrrb, essential TFs such as Sox2, Cdx2, Eomes and Elf5 that sustain TS cell self-renewal.

To account for possible off-target effects of DES treatment, for example, on Esrra and Esrrg, we also performed knockdown (KD) experiments using three short-hairpin RNAs (shRNAs) directed against *Esrrb* (KD-1, KD-2 and KD-3) and two scrambled shRNAs as controls (scr-1 and scr-2). *Esrrb* transcript levels were reduced in the KD-1, KD-2 and KD-3 lines by up to 90% compared with control lines, and these results were also confirmed on the protein level (Fig. 2f,g). We found that depletion of *Esrrb* triggered differentiation despite the presence of Fgf as indicated by the morphological appearance of trophoblast giant cells and loss of proliferative capacity (Fig. 2h). Expression analysis revealed the rapid loss of stem cell markers including *Cdx2*, *Eomes*, *Elf5*, *Nr0b1* and *Bmp4*, and concomitant upregulation of genes associated with trophoblast differentiation including *Syna*, *Gcm1*, *Cdkn1c*, *Prl2c2* (also known as Proliferin=*Plf*) and *Prl3d1* (placental lactogen 1=*Pl1*; Fig. 2g). We confirmed these results at the protein level by using western blot analysis (Fig. 2f). Moreover, this effect was specific to *Esrrb* depletion as co-transfecting the KD-1 shRNA targeted against the 3′-untranslated region with an *Esrrb*-coding region expression construct fully rescued the KD phenotype (Supplementary Fig. 2a,b). These data demonstrate that *Esrrb* is required for TS cell gene expression and self-renewal.

To gain further insights into the cohort of genes regulated by *Esrrb*, we performed an RNA-seq analysis on *Esrrb* KD-1 and KD-2 TS cells 5 days after transfection. Global expression analysis identified 59 genes that were affected by *Esrrb* KD in TS cells (Supplementary Fig. 2c; Supplementary Data 3). Gene ontology (GO) term analysis revealed overrepresentation of processes related to placental development and trophoblast morphology among genes affected by the *Esrrb* KD (Supplementary Fig. 2d,e). In addition, on the global level, downregulated genes contained known TS cell markers including *Eomes*, *Cdx2*, *Nr0b1*, *Id2* and *Sox2*, whereas upregulated genes were highly enriched for factors associated with trophoblast differentiation. These results confirmed that *Esrrb* presides over a network of genes involved in extraembryonic development and specifically in maintenance of the stem cell state within the trophoblast niche.

### Esrrb forms stem cell-type-specific transcriptional networks

**Figure 3:**
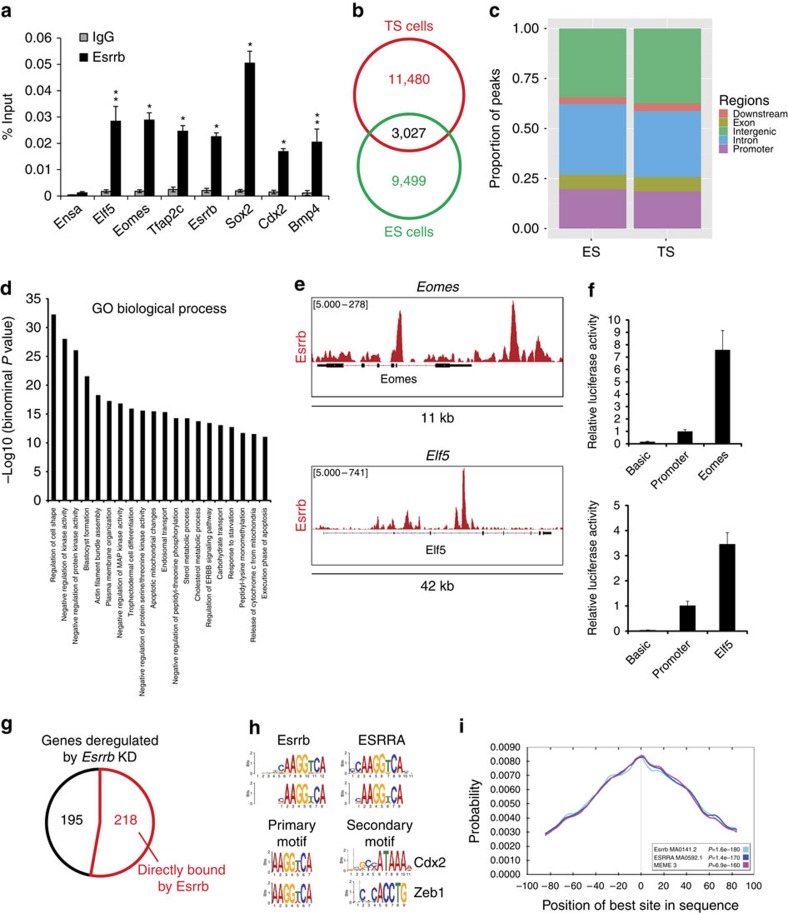
ChIP-seq analysis to identify Esrrb genome-wide occupancy in TS cells. (**a**) Anti-Esrrb ChIP followed by QPCR showing Esrrb binding to TS cell marker genes; the Ensa region serves as a negative control. Bars indicate average of three biological replicates±s.e.m. Statistical test: unpaired *t*-test with Welch’s correction. (**b**) A Venn diagram showing the number of high-confidence Esrrb TS cell-specific peaks identified in five independent biological replicates of ChIP-seq experiments, ES cell-specific peaks^29^ and those overlapping in both stem lines. (**c**) Proportion of Esrrb ChIP-seq peaks overlapping genomic features in TS and ES cells. Peaks overlapping more than one type of genomic region were assigned to regions with the following priority: (1) promoters, (2) exons (3), introns, (4) downstream and (5) intergenic. (**d**) Twenty top terms of the GREAT ontology enrichments for TS cell-specific peaks of Esrrb. (**e**) Examples of Esrrb-binding profiles at the *Eomes* and *Elf5* loci. (**f**) Luciferase reporter analysis of TS cells transiently transfected with putative *Eomes* (Eomes: pGL3-promoter-Eomes) or *Elf5* (Elf5: pGL3-promoter-Elf5) enhancer constructs and controls (basic: pGL3-basic and promoter: pGL3-promoter). Bars show an average of four replicates±s.d., statistical test: unpaired *t*-test with Welch’s correction. (**g**) Pie chart of genes deregulated on *Esrrb* knockdown (posterior probability >0.6) that are also bound by Esrrb. (**h**) Motifs found by MEME and/or DREME and SpaMO to be overrepresented in the Esrrb peaks. (**i**) CentriMO plot of the positional distribution of the best-matched motifs.

To explore whether Esrrb directly regulates the key TS cell genes, we performed chromatin immunoprecipitation (ChIP) followed by QPCR and found extensive binding on putative transcriptional regulatory regions of *Elf5*, *Eomes*, *Esrrb*, *Sox2*, *Bmp4*, *Cdx2* and *Tfap2c* (Fig. 3a). To obtain a comprehensive global overview of the binding sites of Esrrb in TS cells, we carried out ChIP followed by high-throughput sequencing (ChIP-seq) and compared these data to the binding profile of Esrrb in ES cells where it plays a well-appreciated role in maintaining pluripotency^29^. We identified 14507 Esrrb-binding sites in TS cells (Fig. 3b; Supplementary Data 4). Globally, these sites were predominantly found at intronic and intergenic regions (Fig. 3c), similar in feature distribution to that observed in ES cells. However, their precise location exhibited only a partial (3,027) overlap with those in ES cells (Fig. 3b; Supplementary Data 3). The markedly different Esrrb-binding profile between ES and TS cells was exemplified by a significant enrichment of genes involved in trophectodermal differentiation and placental development among the TS cell-specific peaks compared with the ES cell-specific peaks (Fig. 3d; Supplementary Fig. 3a). These results suggest that context-dependent binding of Esrrb is linked to specific developmental processes. Notably, we identified Esrrb binding at principally all known core TS cell genes, including itself, implying that Esrrb has a self-reinforcing function similar to that ascribed to many pluripotency genes in ES cells (Fig. 3e; Supplementary Fig. 3b).

We tested the functionality of the Esrrb-binding sites at *Eomes* and *Elf5*, that is, two of the important TS cell genes we had identified as primary targets of Esrrb by ChIP–QPCR and ChIP-seq, in luciferase assays. Selected regions of both genes stimulated reporter activity (Fig. 3f), and this effect was abolished by either mutating Esrrb-binding sites or by DES treatment (Supplementary Fig. 3c). These results further confirmed that Esrrb directly binds to and regulates *Eomes* and *Elf5* in TS cells. On a more global level, the majority of genes deregulated either on *Esrrb* KD or 24 h DES treatment were directly bound by Esrrb (Fig. 3g; Supplementary Fig. 3d).

To gain better insights into the context-dependent Esrrb binding, we performed *de novo* motif analysis using MEME/DREME followed by Tomtom suits^30^,^31^. In TS cells, similar to ES cells, Esrrb peaks (defined here as ±200 bp around peak summit) were highly enriched in the canonical Esrrb/Esrra-binding motifs, suggesting that the context-dependent binding specificity may rely on other TFs (Fig. 3h). Central motif enrichment analysis^32^ showed centred and symmetrical Esrrb/Esrra motif distribution (Fig. 3i). Space motif analysis (SpaMo) identified, among others, Cdx2 as a secondary motif enriched in a number Esrrb peaks (Fig. 3h). These findings raised the question of whether Cdx2 could potentially recruit Esrrb to TS cell-specific sites and thereby mediate the context-dependent activity of Esrrb in TS versus ES cells.

To examine the functional overlap of genes regulated by Cdx2 and Esrrb, we depleted *Cdx2* in TS cells by shRNA-mediated KD. Expression analysis showed that similar to the *Esrrb* KD, key TS cell markers were downregulated (*Esrrb*, *Eomes* and *Elf5*), whereas differentiation markers were upregulated (Supplementary Fig. 4a). However, when we compared ChIP-seq data sets of Esrrb (this study) and Cdx2 (published by Chuong *et al.*^33^), we identified only a small (4.1%) subset of Esrrb peaks that were co-bound by Cdx2 when using the previously published list of 11462 Cdx2-specific peaks (Supplementary Fig. 4b; Supplementary Data 5) and even fewer (<1%) when applying the identical analysis criteria used in our study on the Cdx2 ChIP-seq data set for peak calling (Supplementary Fig. 4c). This small subset of co-bound loci did not contain any prominent known TS cell genes. To further examine the potential cooperation between Esrrb and Cdx2, we performed co-immunoprecipitation experiments followed by either western blot or mass spectrometry analysis. While we identified a number of Cdx2 interactors including Tead4, Eomes and Tfcp2, we were unable to detect Esrrb (Supplementary Fig. 5a–d). Thus, despite the fact that Esrrb and Cdx2 depletion interferes with TS cell maintenance, ultimately by affecting a similar subset of genes, we found neither that Cdx2 accompanied Esrrb binding at the key TS cell loci nor that they interacted at the protein level. Thus, in line with the evidence that *Cdx2* is not among the early responders on 24 h DES treatment, it is likely that Cdx2 and Esrrb function in parallel pathways to regulate the stem cell state of TS cells.

### Epigenetic protein interaction network of Esrrb in TS cells

Esrrb is part of a large protein network in ES cells that is required to maintain pluripotency^22^,^23^. Two main classes of interactors dominate this network: (i) epigenetic protein complexes that remodel or modify nucleosomes (for example, SWI/SNF, NuRD, p400) and (ii) TFs/cofactors that can directly stimulate RNAPII recruitment and activation (Mediator complex, components of transcriptional machinery, TFs)^22^,^23^. We thus set out to explore which of these distinct mechanisms of Esrrb-mediated control of gene expression were predominant in TS cells.

**Figure 4:**
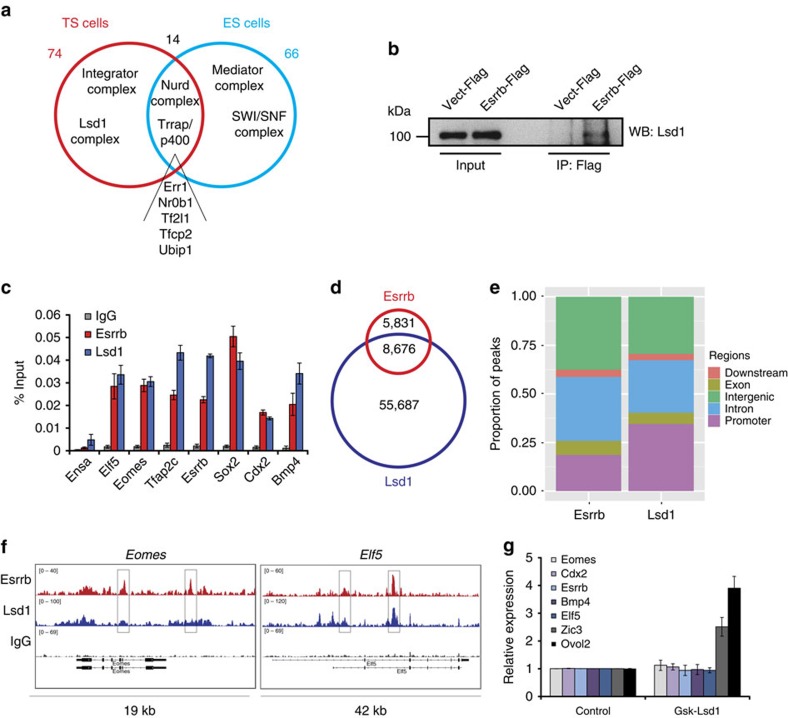
Lsd1 interacts with Esrrb in TS cells. (**a**) Venn diagram showing total numbers and highlighted examples of TS- and ES-cell-specific, as well as shared, Esrrb interactors. Esrrb-interacting proteins as identified using mass spectrometry analysis. High-confidence (that is, present in both Esrrb samples and absent or showing negligible amount in vector control samples) hits are shown. (**b**) Esrrb-3xFlag immunoprecipitates analysed by western blot probed with anti-Lsd1 antibody independently confirms the interaction between Esrrb and Lsd1. (**c**) ChIP–QPCR showing co-occupancy of Esrrb and Lsd1 at the key TS cell marker genes. Ensa serves as a negative control. Bars represent average of three biological replicates±s.e.m. (note that Esrrb data are the same as in Fig. 3a). (**d**) Venn diagram depicting the overlap between Esrrb and Lsd1 ChIP-seq peaks in TS cells. (**e**) Proportion of Esrrb and Lsd1 ChIP-seq peaks overlapping genomic features in TS cells. Peaks overlapping more than one type of genomic region were assigned to regions with the following priority: (1) promoters, (2) exons, (3) introns, (4) downstream and (5) intergenic. (**f**) Esrrb- and Lsd1-binding profiles at the *Elf5* and *Eomes* loci. (**g**) RT–QPCR expression analysis of TS cell markers on 48 h treatment with Lsd1 inhibitor GSK-Lsd1 in TS cells grown in stem cell conditions. *Ovol2* and *Zic3* serve as positive controls as they were reported to be upregulated on Lsd1 depletion^36^.

To identify the interaction partners of Esrrb that are specific to TS cells, we established a TS cell line expressing modest levels of C-terminally 3xFlag-tagged Esrrb. RT–QPCR and western blot analysis showed that the Esrrb-Flag TS cell line was indistinguishable from the vector control (Supplementary Fig. 6a,b). Next, we purified Esrrb-bound proteins in mild conditions, identical to those employed in ES cells^23^. Using an unbiased protein identification approach using liquid chromatography-tandem mass spectrometry (LC-MS/MS), we found Esrrb (29 and 30 unique peptides, protein annotated as ‘ERR2’) in addition to numerous high-confidence interaction partners in several independent experiments (Table 1, Supplementary Data 6). Among these, we detected a number of epigenetic complexes that were previously identified as parts of the Esrrb interactome in ES cells including multiple subunits of NuRD, p400/Trrap and Mll/Trx (Fig. 4a)^23^. Interestingly, we never detected any component of the SWI/SNF complex, another prominent interactor in ES cells that is essential for early embryogenesis^23^,^34^.

Instead, the TS cell-specific Esrrb protein network included components of the lysine-specific demethylase 1 (Lsd1, also known as Kdm1a) complex (Table 1, Supplementary Data 6). Lsd1 is a histone demethylase that selectively removes mono- and dimethyl groups from either lysine 4 of histone H3 (H3K4) or H3K9 (ref. 35). Intriguingly, recent evidence points to an important function of Lsd1 in maintaining the TS cell state by preventing early onset of differentiation^36^. We confirmed the presence of Lsd1 in Esrrb immunoprecipitates by immunoblotting (Fig. 4b); we also performed a reciprocal identification of Lsd1 interactors by rapid immunoprecipitation mass spectrometry of endogenous proteins (RIME)^37^. The LC-MS/MS analysis identified Esrrb as one of the Lsd1 protein interactors in TS cells in addition to other Lsd1-specific interacting TFs (for example, Scmbt2 or Ap2c (=Tfap2c)) and chromatin-modifying complexes (for example, subunit of the FACT complex; Supplementary Table 1; Supplementary Data 7). Taken together, these results suggested that Esrrb operates in distinct protein complexes that exert specific functions in TS cells and more general functions shared with ES cells.

We then sought to investigate in more detail the cooperative function between Lsd1 and Esrrb. For this purpose, we performed Lsd1 ChIP–QPCR and ChIP-seq analyses in TS cells and compared this with the Esrrb occupancy profiles. Importantly, Lsd1 bound to the core set of Esrrb targets including *Elf5*, *Eomes*, *Bmp4* and *Sox2* (Fig. 4c); globally 60% of Esrrb peaks were co-occupied by Lsd1 (Fig. 4d–f; Supplementary Data 8) and co-bound loci were associated with a significant proportion of genes deregulated on Esrrb inhibition or KD (Supplementary Fig. 7a). However, when we specifically inhibited Lsd1, genes involved in onset of differentiation were upregulated (including *Ovol2* and *Zic3*) but expression of the key TFs controlling TS cell self-renewal was not, or only mildly, affected (Fig. 4g). This result is in line with previous reports suggesting a role of Lsd1 primarily in regulating differentiation genes^36^, as also supported by Lsd1’s broad expression pattern within the entire trophoblast compartment (Supplementary Fig. 7b).

### Transcriptional protein interactome of Esrrb in TS cells

**Figure 5:**
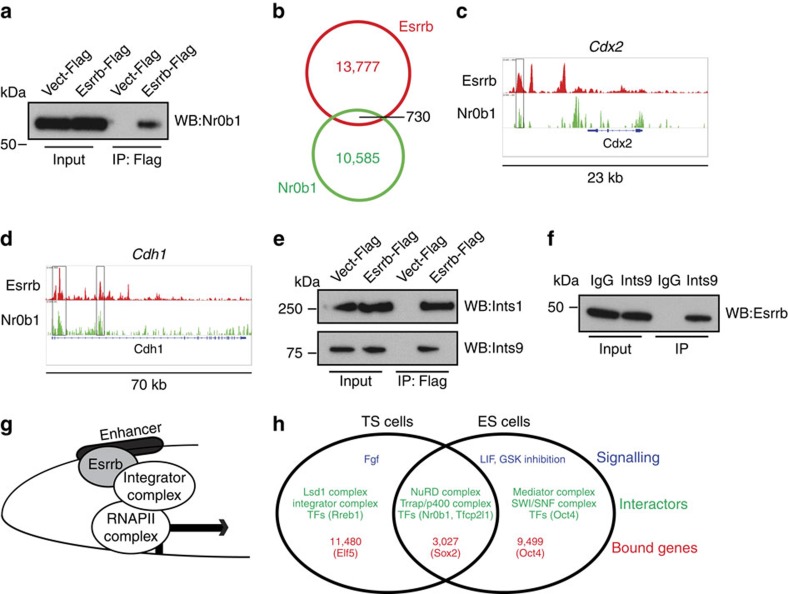
Nr0b1 (=Dax1) and Integrator as Esrrb interactors in TS cells. (**a**) Esrrb-3xFlag immunoprecipitates analysed using western blot probed with anti-Nr0b1 antibody (Supplementary Fig. 10d). (**b**) Venn diagram showing the overlap between Esrrb and Nr0b1 ChIP-seq peaks in TS cells. (**c**) Esrrb- and Nr0b1-binding profiles at the *Cdx2* and (**d**) *Cdh1* loci. (**e**) Esrrb-3xFlag immunoprecipitates analysed using western blot probed with anti-Integrator1 (Ints1) and anti-Integrator 9 (Ints9) antibodies, confirming the prominent interaction between Esrrb and Integrator complex components (Supplementary Fig. 10e). (**f**) Endogenous anti-Ints9 immunoprecipitates analysed by western blot with anti-Esrrb antibody shows that Esrrb interacts with Ints9 (Supplementary Fig. 10f). (**g**) Context-dependent function of Esrrb in TS cells may be in part mediated by the interaction with the Integrator complex. (**h**) Comparison of Esrrb-centred self-renewal networks in TS cells and ES cells. Although Esrrb expression is driven by distinct signalling pathways in TS (Fgf/Mek) and ES (LIF/Gsk inhibition) cells, some interacting partners are shared (for example, transcription factors (TFs) Nr0b1, Tfcp2lf1 and the NuRD complex), whereas others are stem cell-specific (for example, Integrator or Mediator complexes). Similarly, Esrrb commonly binds a shared set of genes in both stem cell types, in addition to TS- and ES-cell-specific targets.

Besides interactors involved in epigenetic regulation of transcription, we identified also TFs and cofactor complexes that directly interact with RNAPII (Table 1; Fig. 4a). Similar to some shared epigenetic complexes, we found that the TFs Nr0b1, Esrra, Tf2l1, Zfp462 and others overlapped with the Esrrb interactome in ES cells, thereby further validating our immunoprecipitation (IP) LC-MS/MS analysis (Fig. 4a). Since Nr0b1 has been found to have an important role in ES cell self-renewal, we confirmed by co-immunoprecipitation that it also interacts with Esrrb in TS cells (Fig. 5a). ChIP-seq analysis for Nr0b1 in TS cells showed binding overlap with Esrrb on a subset of essential TS cell-specific (for example, *Cdx2* and *Tfap2*) and general developmental loci (*Lin28a* and *Cdh1*; Fig. 5b–d; Supplementary Fig. 8a; Supplementary Data 8). As with Esrrb before, we observed that Nr0b1 binding in TS and ES cells showed a small overlap, with only 52 Esrrb/Nr0b1 co-bound regions shared between ES and TS cells (Supplementary Fig. 8b, Supplementary Data 8). These detailed novel data on the context-specific wiring of transcriptional networks are supported also by the limited overlap of Tfcp2l1, another TF that complexes with Esrrb in both TS and ES cells, with Esrrb TS cell peaks (Supplementary Fig. 8c; Supplementary Data 8).

Intriguingly, in contrast to the Esrrb interactome in ES cells^23^, we never detected components of the prominent RNAPII-associated complex Mediator as an Esrrb interactor in TS cells. This finding prompted us to search for alternative explanations of Esrrb-mediated RNAPII recruitment and activation at its target genes involved in TS cell self-renewal. Strikingly, instead of components of the Mediator complex we identified four subunits of another key RNAPII cofactor complex named Integrator (Table 1). We validated expression of some Integrator complex components as well as other identified Esrrb interactors in ES and TS cells and observed similar levels despite context-specific interactions (Supplementary Fig. 9). We also confirmed the interaction of Integrator components with Esrrb by co-immunoprecipitation (Fig. 5e,f). Until recently, the Integrator complex was implicated in small nuclear RNA transcription but a recent study found that it also functions in Egf-mediated transcriptional activation of immediate-early-response genes^38^,^39^. This important finding may explain how Esrrb attracts the transcriptional machinery in the absence of the interaction with Mediator in TS cells (Fig. 5g).

In summary, our results provide comprehensive insights into the stem cell-type-specific regulation and function of Esrrb, suggesting an exciting mechanism of how Fgf via Esrrb can rapidly and specifically impact on the transcription of key genes controlling self-renewal of TS cells (Fig. 5h).

## Discussion

Esrrb is known to play a central role in maintaining pluripotency of ES cells by acting in concert with various other key pluripotency genes. Despite this, mouse mutants deficient for *Esrrb* die of a trophoblast defect that can be rescued by tetraploid aggregation experiments, thus definitively ruling out a contributing defect intrinsic to the embryo proper^12^. Although it has been demonstrated that *Esrrb* is required for early trophoblast development, the function of *Esrrb* in TS cells has not yet been elucidated. Here we show that Esrrb establishes highly stem cell-type-specific functional networks both at the level of chromatin occupancy as well as at the level of protein–protein interactions. While some overlap in binding partners and target gene profile is observed between ES and TS cells that may confer more generic ‘stemness’ functions, we here show that Esrrb exerts lineage-specific pivotal roles in the TS cell compartment. Our data demonstrate that, in striking contrast to the situation in ES cells, *Esrrb* is an immediate target of Fgf/Mek signalling in TS cells and in turn directly activates key TS cell genes. To decipher the mechanism whereby Esrrb regulates TS cell-specific transcriptional regulation, we identified the Esrrb protein interactome—the first of its kind in TS cells to date.

Several lines of evidence suggest that Esrrb is the main mediator of Fgf-driven Erk signalling in TS cells. First, Esrrb is rapidly downregulated on Mek inhibition identifying it as a direct target of the immediate-early Mek/Erk response. Second, the overlap of genes that are misregulated on short-term inhibition of Fgf–Erk signalling (PD03) and Esrrb (DES) includes key TS cell regulators such as *Sox2*, *Eomes*, *Cdx2* and *Elf5*. Third, a great proportion of genes that were deregulated by either Esrrb KD or DES treatment are bound by Esrrb, strongly supporting their direct regulation. Indeed, we confirmed such a direct transcriptional control function of Esrrb at the *Eomes* and *Elf5* loci, where mutagenesis of Esrrb-binding sites in putative enhancer regions abolished luciferase reporter activity. This effect was apparent despite the presence of Fgf signalling demonstrating that Esrrb binding is vital for activation of *Elf5* and *Eomes*. Thus, Esrrb is an essential mediator of Fgf–Erk signalling that induces *Elf5* and *Eomes* expression. Taken together, our data show that Fgf–Erk and Esrrb constitute the major axis controlling critical TS cell genes.

If Esrrb has diverse functions in different developmental contexts, we would expect that it binds to and regulates different genes in these settings. Indeed, we found that there is only a partial overlap of sites bound by Esrrb in ES and TS cells suggesting that some functions of Esrrb might be conserved (for example, driving self-renewal and proliferation) while others might be divergent. This insight led to the crucial question about protein interaction partners that mediate the general and specific functions of Esrrb in TS versus ES cells. To date, we are lacking protein interactomes in TS cells that would clarify whether the same general factors and mechanisms drive self-renewal in embryonic and extraembryonic stem cells. In this study we provide a comprehensive analysis of the Esrrb, Lsd1 as well as the Cdx2 binding partners in TS cells as a key resource to elucidate their mechanistic roles in stemness and trophoblast development. Similar to Esrrb-interacting proteins in ES cells, we identified two separate classes of Esrrb interactors in TS cells: (i) epigenetic regulators that remodel and modify chromatin and (ii) regulators that can interact directly with the transcriptional machinery. Importantly, we found that, while some of these interactors in TS cells overlap with ES cells, others do not, further suggesting both general and specific mechanisms of Esrrb action in distinct stem cell types.

One of the proteins identified as a TS cell-specific Esrrb interactor was the lysine-specific demethylase Lsd1. In ES cells, Lsd1 occupies enhancers of active genes critical for pluripotency. On differentiation, Lsd1 decommissions these enhancers ensuring the shutdown of the pluripotency programme^40^. In contrast, in TS cells, it has been shown that the transcription of stem cell marker genes *Cdx2* and *Eomes* is reduced considerably faster in the absence of *Lsd1* than in controls on induction of differentiation, in line with the observation that *Lsd1*-depleted TS cells exhibit a lowered threshold for differentiation onset^36^. Thus, although depletion^36^ or inhibition of Lsd1 has no clear-cut effect on TS cell marker silencing in stem cell conditions (Fig. 4g), it appears that Esrrb and Lsd1 cooperatively promote the ‘naive’ TS cell state to maintain a fine-tuned balance of gene transcription at joint TS cell target genes.

Besides epigenetic regulators, we identified numerous TFs that interact with Esrrb in TS cells. One of these factors is Nr0b1 (=Dax1), which associates with Esrrb also in ES cells^23^,^41^,^42^. Nr0b1 is part of the ES cell self-renewal network where it interacts with Oct4 and gets recruited to Oct4/Sox2-binding sites^23^,^42^. However, we discovered that similar to Esrrb, Nr0b1 does not show an extensive binding overlap between TS and ES cells, again underpinning the finding that, although both TFs are shared between ES and TS cells, they exert largely divergent functions depending on stem cell type. This raises the question about how the context-dependent recruitment of Esrrb and Nr0b1 to distinct sites is achieved in different stem cells. Regarding Esrrb, Cdx2, as a key TS cell regulator, is an obvious candidate for this role. This notion is further supported by our findings that similar genes are downregulated on Esrrb and Cdx2 depletion. However, we could not detect an extensive overlap between published Cdx2 (ref. 33) and our Esrrrb ChIP-seq-binding profiles, and neither did we observe a direct interaction between these two factors at the protein level. We did, however, identify other prominent Cdx2 interactors including Tead4 and Eomes, thus strongly validating our approach. Although Esrrb and Cdx2 ultimately co-regulate, directly or indirectly, a similar set of target genes, it is therefore likely that both TFs function in parallel pathways to regulate the stem cell state of TS cells. Taken together, these findings provide new and comprehensive insights into the TF interaction network that governs TS cell self-renewal and identity. It will be important to elucidate in the future how this network exerts specificity in TS cells with partially shared components present also in ES cells.

In fact, our comprehensive identification of interaction partners may provide first leads into how this context-dependent wiring of transcriptional networks is achieved, by revealing association with distinct components of the core transcriptional machinery depending on stem cell type. In ES cells, Esrrb was identified as being uniquely associated with the RNAPII complex and numerous subunits of the Mediator complex^23^, indicating a critical role for Esrrb in transcriptional activation. The Mediator complex is a multifunctional RNAPII-associated scaffold that is required for mRNA transcription at different stages of the process. The interaction with TFs is crucial for recruitment and specificity in response to signalling^43^. In TS cells we did not detect an interaction between Esrrb and the Mediator complex raising the question of an alternative way to stimulate transcription. Instead, we identified numerous subunits of the Integrator complex interacting with Esrrb. Although the Integrator complex has been implicated mostly in the transcription of small nuclear RNAs^38^, a recent study demonstrated its involvement in both initiation and release from pausing of RNAPII during mRNA transcription^39^. Intriguingly, this mechanism was demonstrated for early-response genes that are activated by Egf. Since Fgf has also a very rapid impact on transcription of some key genes, notably *Esrrb*, in TS cells, this raises the exciting possibility that Esrrb activates transcription by association with the Integrator complex and release of RNAPII from pausing (Fig. 5g). This would suggest that not only specific signals and TFs shape self-renewal and identity of different stem cell types but that general mechanisms of transcriptional control also contribute to confer stem cell specificity.

Taken together, we demonstrate here an essential TS cell-specific role of Esrrb and provide key insights into mechanisms of Fgf–Erk-mediated self-renewal in TS cells.

## Methods

### Tissue culture and transfections

Mouse TS cells (blastocyst-derived TS EGFP line, a kind gift of Dr Janet Rossant, Toronto, Canada), proven to exhibit full developmental competence as they colonize all trophoblast layers in chimeras, were cultured as described previously^44^. Briefly, TS cells were grown in a standard TS medium (RPMI 1640 supplemented with 20% fetal calf serum, 2 mM L-Glutamine, 2 mM sodium pyruvate and 100 mM 2-mercaptoethanol) containing 70% mouse embryonic fibroblast -conditioned medium and 25 ng ml^−1^ Fgf2 and 1 μg ml^−1^ heparin. Cells were split every third day using trypsin. Transfections were performed for 6 h in OptiMEM media supplemented with Fgf2 and heparin using 1% Lipofectamine 2000 (Life Technologies) on nonadherent dishes. After 24 h, cells were selected with 300 μg ml^−1^ G418. Inhibitors used were as follows: 1 μM LDN 193189 trihydrochloride (Axon, 1509); 2 μM endo-IWR-1 (Tocris, 3532); 2 μM PD0325901; 3 μM CHIR99021; 50 nM Gsk-Lsd1 (N-[(1R,2S)-2-phenylcyclopropyl]-4-piperidinamine, dihydrochloride), kindly provided by the Structural Genomics Consortium (http://www.thesgc.org); 10 μM SB431542; and 15 μM DES (Sigma).

### Chromatin immunoprecipitation

Immunoprecipitations were carried out as described^45^. Briefly, cells (1–2 × 10^8^) were fixed in 2 mM Di(N-succinimidyl) glutarate (DSG) (80424, Sigma) in PBS at room temperature (RT) for 45 min. After washing in PBS, cells were fixed again in 1% formaldehyde in TS base media at RT for 12 min. Fixation was stopped by adding glycine to a final concentration of 0.125 M. Cells were washed twice in PBS and resuspended in wash buffer 1 (10 mM Hepes pH 7.5, 10 mM EDTA, 0.5 mM EGTA and 0.75% Triton X-100) and incubated at 4 °C for 10 min. After pelleting, cells were resuspended in wash buffer 2 (10 mM Hepes pH 7.5, 200 mM NaCl, 1 mM EDTA and 0.5 mM EGTA) and incubated at 4 °C for 10 min. After pelleting, cells were lysed in the lysis/sonication buffer (150 mM NaCl, 25 mM Tris pH 7.5, 5 mM EDTA, 0.1% Triton, 1% SDS and 0.5% sodium deoxycholate) with complete protease inhibitors (Roche) on ice for 30 min. Chromatin was sonicated 30 s on/30 off for 25–30 cycles using the BioRuptor (Diagenode) to the average 300-bp fragments. Chromatin was diluted 1/10 with the dilution buffer (150 mM NaCl, 25 mM Tris pH 7.5, 5 mM EDTA, 1% Triton X-100, 0.1% SDS and 0.5% sodium deoxycholate) containing complete protease inhibitors. Protein G magnetic Dynabeads (10004D, Invitrogen) were blocked with 1 mg ml^−1^ BSA and tRNA at 4 °C for 1 h and washed with buffer A (150 mM NaCl, 25 mM Tris pH 7.5, 5 mM EDTA, 1% Triton X-100, 0.1% SDS and 0.5% sodium deoxycholate). Chromatin was pre-cleared with pre-blocked beads at 4 °C for 1 h. Three hundred and fifty micrograms of chromatin and ten micrograms of antibody (mouse anti-Esrrb (Perseus Proteomics PP-H6705-00), mouse normal IgG (Santa Cruz sc-2025), rabbit anti-Nr0b1 (Santa Cruz sc-841X) and rabbit normal IgG (Santa Cruz sc-2027X) were used per each IP. IP was performed overnight at 4 °C with rotation. Pre-blocked magnetic beads were added next morning for 7–8 h. Beads were washed at 4 °C with buffer A (150 mM NaCl, 25 mM Tris pH 7.5, 5 mM EDTA, 1% Triton X-100, 0.1% SDS and 0.5% sodium deoxycholate) three times, buffer B (50 mM Tris pH 8.0, 500 mM NaCl, 0.1% SDS, 0.5% sodium deoxycholate, 1% NP-40 and 1 mM EDTA), buffer C (50 mM Tris pH 8.0, 250 mM LiCl, 0.5% sodium deoxycholate, 1% NP-40 and 1 mM EDTA) and rinsed with TE buffer. DNA was eluted from beads in the elution buffer (1% SDS, 0.1 M NaHCO_3_). Samples were treated with RNAse A and Proteinase K and reverse-crosslinked overnight at 65 °C. DNA was phenol–chloroform-extracted, chloroform-extracted and EtOH/Glyco blue-precipitated (for QPCR analysis) or purified on the PCR purification columns (Qiagen; for ChIP-seq libraries). To generate a library, DNA from four IPs was pooled and the NEB Next DNA Library Prep Master Mix (NEB E6040) was used according to the manufacturer’s instructions. Libraries were amplified using 18 PCR cycles, purified using Agencourt AMPure XP SPRI beads (Beckman Coulter, A63881) and size-selected on an agarose gel. The DNA was extracted using a Qiaquick gel extraction kit (Qiagen) and its concentration determined using the KAPA Illumina SYBR Universal Lid Q Kit (KAPA Biosystems KK4824) and Bioanalyzer 2100 system (Agilent). Libraries were sequenced on Illumina HiSeq1000 sequencer.

The raw reads were trimmed to remove adapter sequences (minimum overlap required of 3 bp) and bad-quality bases at the end of each read using Trim Galore (http://www.bioinformatics.babraham.ac.uk/projects/trim_galore). All reads were aligned to the mouse genome (GRCm38) with Burrows-Wheeler alignment (BWA)^46^ using default options. Peak calling was performed with MACS2 (ref. 47) using only unique mapping and non-duplicated reads from each ChIP sample and a single pooled IgG control. To combine the results from the five replicates without pooling, we used the irreproducible discovery rate (IDR) approach developed by the ENCODE project^48^.

Data for Esrrb ChIP-seq binding in ES cells from ref. 29 (SRX000542 and SRX000543) were downloaded from the European Nucleotide Archive. The publicly available data consisted of four replicate experiments. Fastq files were used as provided without base-trimming, while alignment, peak calling and IDR analysis were performed in the same way as for the in-house samples.

Annotation of binding sites according to genomic features was performed by overlapping the sites with Ensembl v77 annotations. Binding sites within −2,500 and +500 bp from transcription start sites were classified as overlapping ‘promoters’. Binding sites falling between the transcription end site and +2,500 bp downstream of the end site were classified as overlapping ‘downstream’ sites. Binding sites falling outside gene, promoter and downstream sites were classified as ‘intergenic’. Since some peaks overlap multiple annotations, these were disambiguated with the following priority: (1) promoters, (2) exons, (3) introns, (4) downstream, (5) intergenic. Motif analysis was performed with MEME-chip^49^ using the JASPAR_CORE_2014_vertebrate database, searching for zero or one occurrence of the motif per peaks and with a maximum number of motifs discovered by MEME of 12.

Functional annotation of genes associated with peak regions was performed using GREAT^50^ with the whole mouse genome as background.

### RT–QPCR

RNA was isolated using the RNeasy kit (Qiagen) and DNAseI-treated with the TURBO DNA-free kit (Life Technologies AM1907) according to the manufacturer’s instructions. cDNA was synthesized using 3.5 μg RNA primed with random hexamers according to the RevertAid H Minus M-MuLV Reverse Transcriptase protocol (Thermo Scientific EP0451). DNA was diluted and QPCR performed using SYBR Green Jump Start Taq Ready Mix (Sigma S4438), on a Bio-Rad CFX96 thermocycler. Primer pairs are provided in Supplementary Table 2.

### RNA KD

RNA KD experiments were performed using the pSuper-neo system. Oligos (see Supplementary Table 3 for shRNA sequences) were cloned into BglII/XhoI sites. TS cells were transfected with 4.5 μg of plasmid and selected after 24 h with 600 μg ml^−1^ G418.

### Western blot analysis

Whole-cell extracts were prepared with TG buffer (20 mM Tris-HCl pH 7.5, 137 mM NaCl, 1 mM EGTA, 1% Triton X-100, 10% glycerol and 1.5 mM MgCl_2_) supplemented with protease inhibitor cocktail (Roche) and phosphatase inhibitors (50 mM NaF and 1 mM Na_3_VO_4_). Nuclear extracts were prepared with hypotonic buffer 10 mM Hepes pH 7.9, 1.5 mM MgCl_2_, supplemented with protease inhibitor cocktail (Roche). After centrifugation at 10,000*g* for 1 min, nuclear pellets were extracted with 10 mM Hepes pH 7.9, 400 mM NaCl, 10 mM KCl, 1.5 mM MgCl_2_, 0.1 mM EDTA and 12.5% Glycerol supplemented with protease inhibitor cocktail (Roche). Protein lysates were resolved using SDS–PAGE and transferred using a Bio-Rad Mini Trans Blot system 170–3,930 on polyvinylidene difluoride membrane (Immobilon-P, Millipore). Membranes were blocked with 5% milk powder and incubated with specific primary antibodies overnight at 4 °C (1:1,000 anti-Cdx2 (Biogenex MU392A-UC), 1:500 anti-Elf5 (Santa Cruz sc-9645), 1:750 anti-Eomes (Abcam ab23345), 1:1,000 anti-anti-Esrrb (Perseus Proteomics PP-H6705-00), 1:5,000 anti-tubulin (Abcam ab6160), 1:1,000 anti-Int1 (Bethyl Laboratories A300-361A), 1:1,000 anti-Ints9 (Bethyl Laboratories A300-412A), 1:2,000 anti-Flag (SIGMA F1804), 1:1,000 anti-Lsd1 (Abcam ab17721), 1:2,000 anti-Nr0b1 (Santa Cruz sc-841X), 1:750 mouse, anti-phospho Erk1/2 (Cell Signal. 9106), 1:1,000 mouse anti-Erk1/2 (BD 610031), 1:1,000 mouse anti-Oct4 (Santa Cruz sc-5279), followed by horseradish peroxidise-conjugated secondary antibodies (anti-rabbit (Bio-Rad 170–6515), anti-rat (GE Healthcare NA935), anti-mouse (Bio-Rad 170–6516), anti-goat (Abcam ab6885), all 1:2,000). Detection was carried out with enhanced chemiluminescence reaction (GE Healthcare RPN2209) on standard X-ray films. All antibodies are listed in Supplementary Table 5.

### Immunostaining

Cells were fixed in 4% paraformaldehyde/PBS for 20 min at 4 °C, permeabilized and blocked for 30 min in 0.5% bovine serum albumin and 0.1% Triton X-100 in PBS. The following primary antibodies with given dilutions were used: anti-Cdx2 1:500 (Biogenex MU392A-UC), anti-Elf5 1:200 (Santa Cruz sc-9645) and anti-Eomes 1:400 (Abcam ab23345). Alexa Fluor-conjugated secondary antibodies (Life Technologies) were applied at 1:1,000 in 0.5% bovine serum albumin and 0.1% Tween-20 in PBS (PBT-BSA) blocking solution. Cells were counterstained with 4,6-diamidino-2-phenylindole (DAPI) and imaged using a Zeiss LSM700 confocal microscope with the ZEN software.

For immunofluorescence staining of mouse conceptuses, E6.5 implantation sites of wt (C57BL/6Babr × CBA) F1 intercrosses were dissected, counting the day of the vaginal plug as E0.5, and processed for routine paraffine histology. All animal experiments were conducted in full compliance with UK Home Office regulations and with approval of the local animal welfare committee at The Babraham Institute, and with the relevant project and personal licences in place. Sections (7 μm) were deparaffinized, boiled for 30 min in 10 mM sodium citrate pH 6.0 or 1 mM EDTA pH 7.5, 0.05% Tween-20 and blocked with PBT-BSA. Primary antibodies and dilutions used were as follows: mouse anti-Esrrb 1:200 (R&D Systems H6707), rabbit anti-Nr0b1/Dax1 1:200 (Santa Cruz sc-841), rabbit anti-Lsd1 1:100 (Abcam ab17721) and goat anti-Sox2 1:100 (R&D Systems AF2018). Primary antibodies were detected with appropriate secondary AlexaFluor 488, 568 or 647 antibodies, counterstained with DAPI and observed using an Olympus BX41 or BX61 epifluorescence microscope. All antibodies used are listed in Supplementary Table 5.

### Co-immunoprecipitation

Esrrb-coding sequence (PiggyBac-Esrrb-ires-Neo, a kind gift from Austin Smith, CSCR, Cambridge, UK) was cloned to result in PiggyBac-CAG-Avi-Esrrb-3xFlag-ires-Neo construct. TS EGFP cells were transfected with the construct along with the empty vector control using Lipofectamine 2000 (Invitrogen), selected with G418 and expanded in 10 15-cm dishes. Co-immunoprecipitation was performed as described before^23^. Cells were washed in PBS, harvested, resuspended in Buffer A (10 mM Hepes pH 7.6, 1.5 mM MgCl_2_ and 10 mM KCl) and disrupted by 10 strokes in dounce homogenizer. Extracts were spun down and the pellet resuspended in Buffer C (20 mM Hepes pH 7.6, 25% Glycerol, 420 mM NaCl, 1.5 mM MgCl_2_ and 0.2 mM EDTA), passed through a 19-G needle and dialysed to Bufffer D (20 mM Hepes pH 7.6, 20% Glycerol, 100 mM KCl, 1.5 mM MgCl_2_ and 0.2 mM EDTA) using dialysis cassettes (Fisher Scientific). Anti-FLAG M2 agarose beads (120 μl; Sigma) equilibrated in buffer D were added to 1.5 ml of nuclear extract in No Stick microcentrifuge tubes (Alpha Laboratories) and incubated for 3 h at 4 °C in the presence of Benzonase (Novagen). Beads were washed five times for 5 min with buffer D containing 0.02%NP-40 (C-100*) and bound proteins were eluted four times for 15 min at 4 °C with buffer C-100* containing 0.2 mg ml^−1^ FLAG-tripeptide (Sigma). Eluates were pooled and analysed using mass spectrometry or western blot.

### Mass spectrometry

Immunoprecipitated proteins from two biological replicates each of Esrrb- and vector-transfected TS cells were run a short distance (∼5 mm) into an SDS–PAGE gel, which was then stained with colloidal Coomassie stain (Imperial Blue, Invitrogen). The entire stained gel pieces were excised, destained, reduced, carbamidomethylated and digested overnight with trypsin (Promega sequencing grade, 10 ng μl^−1^ in 25 mM ammonium bicarbonate) as previously described^51^. The resulting tryptic digests were analysed using LC-MS/MS on a system comprising a nanoLC (Proxeon) coupled to a LTQ Orbitrap Velos Pro mass spectrometer (Thermo Scientific). LC separation was achieved on a reversed-phase column (Reprosil C18AQ, 0.075 × 150 mm, 3 μm particle size), with an acetonitrile gradient (0–35% over 180 min, containing 0.1% formic acid, at a flow rate of 300 nl min^−1^). The mass spectrometer was operated in a data-dependent acquisition mode, with an acquisition cycle consisting of a high-resolution precursor ion spectrum over the *m*/*z* range 350–1,500, followed by up to 20 CID spectra (with a 30-s dynamic exclusion of former target ions). Mass spectrometric data were processed using Proteome Discoverer v1.4 (Thermo Scientific) and searched against the mouse entries in Uniprot 2013.09, and against a database of common contaminants, using Mascot v2.3 (Matrix Science). Quantitative values were calculated with Proteome Discoverer for each identified protein as the average of the three highest peptide ion peak areas. The search results and quantitative values were imported into Scaffold v3.6 (Proteome Software Inc.), which reported a total of 1,249 proteins across the four samples, with a calculated protein false discovery rate of 0.2%. After applying further filters (minimum of two unique peptides per protein with at least one in both biological replicates, ratio of quantitative values >2 for both Esrrb/vector pairs) 90 proteins remained, as shown in Supplementary Data 6.

### RIME

RIME was carried out as described^37^. Briefly, cells were crosslinked in media containing 1% formaldehyde (EM grade; tebu-bio) for 8 min. Crosslinking was quenched by adding Glycine to a final concentration of 0.2 M. The cells were washed with and harvested in ice-cold PBS. The pellet was resuspended in 10 ml of LB1 buffer (50 mM HEPES-KOH (pH 7.5), 140 mM NaCl, 1 mM EDTA, 10% glycerol, 0.5% NP-40 or Igepal CA-630 and 0.25% Triton X-100) for 10 min at 4 °C. Cells were pelleted, resuspended in 10 ml of LB2 buffer (10 mM Tris-HCL (pH 8.0), 200 mM NaCl, 1 mM EDTA and 0.5 mM EGTA), and mixed at 4 °C for 5 min. Cells were pelleted and resuspended in 300 μl of LB3 buffer (10 mM Tris-HCl (pH 8), 100 mM NaCl, 1 mM EDTA, 0.5 mM EGTA, 0.1% Na-deoxycholate and 0.5% N lauroylsarcosine) and sonicated in a water bath sonicator (Diagenode Bioruptor). A total of 30 μl of 10% Triton X-100 was added, and the lysate was centrifuged for 10 min at 20,000 r.c.f. The supernatant was then incubated with 100 μl of magnetic beads (Dynal) prebound with 20 μg of either anti-Lsd1 (ab 17721 Abcam) or anti-IgG (sc-2027 Santa Cruz) antibody, and IP was conducted overnight at 4 °C. The beads were washed 10 times in 1 ml of RIPA buffer and twice in 100 mM ammonium hydrogen carbonate solution. Detailed results including peptide sequences, peptide scores, ion scores, expect values and Mascot scores have been included in Supplementary Data 7.

### RNA-seq

Total RNA was prepared using the RNeasy kit (Qiagen 74104) followed by DNase treatment using the TURBO DNA-free kit (Life Technologies AM1907) according to the manufacturers’ instructions. mRNA was isolated using the Dynabeads mRNA purification kit (Life Technologies 61006) and prepared into an indexed library using the ScriptSeq v2 RNA-Seq Library Preparation Kit (Epicentre SSV21106) according to the manufacturers’ instructions. Libraries were quantified/assessed using both the KAPA Library Quantification Kit (KAPA Biosystems KK4824) and Bioanalyzer 2100 system (Agilent). Indexed libraries were pooled and sequenced with a 100-bp single-end protocol. The raw reads were trimmed to remove adapter sequences (minimum overlap required of 3 bp) and bad-quality bases at the end of each read using Trim Galore (http://www.bioinformatics.babraham.ac..uk/projects/trim_galore). All reads were aligned to version 75 of the Ensembl mouse reference cDNA and ncRNA sequences using Bowtie 1 (ref. 52) allowing for multimapping between reads and transcripts. The MMSEQ gene expression analysis software^53^ was used to estimate gene expression levels. The marginal posterior mean and s.d. of the log expression parameter corresponding to each gene was used as the outcome in a Bayesian model selection algorithm implemented in the MMDIFF software^54^. In the *Esrrb* KD analysis, differential expression between biological replicates of the *Esrrb* KD (2 × KD-1, 2 × KD-2) and control (2 × scr-1, 1 × scr-2) was determined by comparing a baseline model with a single mean log expression parameter to an alternative model in which the two conditions have different means. We specified a prior probability for the alternative model of 0.1 and declared as differentially expressed those genes for which the posterior probability of the alternative model exceeded 0.6. In the PD03 RNA-seq analysis, two biological replicates were used for each condition (2 × ctrl 3 h, 2 × ctrl 24 h, 2 × PD03 3 h and 2 × PD03 24 h). In this analysis, we compared a baseline model specifying a single mean log expression parameter for all samples and an alternative model specifying a fold change parameter representing the difference in means between the four control samples and the four PD03 samples and a different fold change parameter representing the difference in means between the two PD03 3 h samples and the two PD03 24 h samples. The following design matrices were used to compare the baseline and alternative models, respectively:




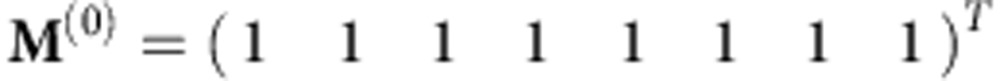







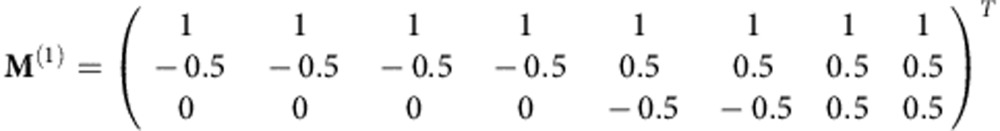




where the first four rows correspond to control samples, the next two rows correspond to PD03 3 h samples and the last two rows correspond to PD03 24 h samples. Note that the matrices have been transposed to optimize the use of space. In this analysis, a high-confidence set of genes misregulated by the PD03 treatment was established by selecting genes for which the posterior probability of the more complex model exceeded 0.95. Within this set, genes for which the absolute estimated 3 h fold change was greater than 1 were labelled ‘early responders’, while the others were labelled ‘gradual responders’. A similar model comparison was used to analyse the DES data comprising three control samples, four samples taken after 24 h of DES treatment and three samples taken 4 days after DES treatment. In both analyses, the prior distributions for the intercepts and regression coefficients were set as described previously^53^. For enrichment analysis of differentially expressed genes, we used MouseMine (www.mousemine.org), setting a Holm–Bonferroni-corrected *P* value threshold of 0.05.

### Luciferase assays

Putative wt or mutated Eomes and Elf5 enhancers were cloned into the BamHI site of the pGL3-promoter vector (Promega) and co-transfected with Renilla plasmid into the TS EGFP line. Site-directed mutagenesis was performed using the QuikChange Site-Directed Mutagenesis Kit (Agilent Technologies) following the manufacturer’s instructions. Control lines were generated by co-transfection of Renilla with either pGL3-promoter or pGL3-basic vectors (Promega). Cells were harvested 48 h after transfection and luciferase activity was measured with the Dual-Luciferase Reporter Assay kit (Promega E1910) following the manufacturer’s instructions using a Promega GloMax 96-well luminometer running Glomax software. Firefly activity was normalized to Renilla luciferase activity values, which are represented with their s.d. Primer sequences are provided in Supplementary Table 4.

## Additional information

**How to cite this article:** Latos, P. A. *et al.* Fgf and Esrrb integrate epigenetic and transcriptional networks that regulate self-renewal of trophoblast stem cells. *Nat. Commun.* 6:7776 doi: 10.1038/ncomms8776 (2015).

## Supplementary Material

Supplementary Information Supplementary Figures 1-10, Supplementary Tables 1-5 and Supplementary References

Supplementary Data 1 Genes affected by PD0325901 treatment of TS cells

Supplementary Data 2 Genes affected by DES treatment of TS cells

Supplementary Data 3 Genes differentially expressed upon Esrrb knockdown in TS cells

Supplementary Data 4 Genes bound by Esrrb in ES cells

Supplementary Data 5 Genes bound by Cdx2 in TS cells (based on Chuong et al., 2013)

Supplementary Data 6 Proteins identified by anti-Esrrb mass spectrometry

Supplementary Data 7 Proteins identified in the anti-Lsd1 RIME

Supplementary Data 8 Genes bound by Tcfcp2l1 in ES cells (based on Chen et al., 2008)

## Figures and Tables

**Table 1:** TS cell-specific Esrrb interactome.

**Identified Proteins**	**Accession**	**Score**		**Number of unique peptides**		**% Coverage**	
		**Esrrb-2**	**Esrrb-1**	**Esrrb-2**	**Esrrb-1**	**Esrrb-2**	**Esrrb-1**
Steroid hormone receptor ERR2	ERR2_MOUSE	1362	2029	30	29	65	62
							
*Lsd1 complex*							
Lysine-specific histone demethylase 1A	KDM1A_MOUSE	291	150	27	10	48	21
REST co-repressor 1	RCOR1_MOUSE	202	96	7	3	20	14
Histone–lysine N-methyltransferase EHMT1	EHMT1_MOUSE	79	93	15	5	13	5
C-terminal-binding protein 1	CTBP1_MOUSE	53	28	5	2	17	3
Histone–lysine N-methyltransferase EHMT2	A2CG76_MOUSE	51	38	15	4	17	6
							
*MII complex*							
Sentrin-specific protease 3	SENP3_MOUSE	508	249	21	16	42	32
Ribosomal biogenesis protein LAS1L	LAS1L_MOUSE	442	550	24	26	34	38
Host cell factor 1	HCFC1_MOUSE	123	96	14	7	9	5
Set1/Ash2 histone methyltransferase complex subunit ASH2	ASH2L_MOUSE	110	23	7	1	17	3
							
*Integrator complex*							
Integrator complex subunit 7	INT7_MOUSE	322	248	14	16	21	24
Integrator complex subunit 6	INT6_MOUSE	312	318	16	18	23	28
Integrator complex subunit 10	INT10_MOUSE	91	140	5	11	9	20
Integrator complex subunit 9	INT9_MOUSE	62	127	5	10	13	21
							
*p400 complex*							
E1A-binding protein p400	EP400_MOUSE	236	46	17	2	12	1
DNA methyltransferase 1-associated protein 1	DMAP1_MOUSE	100	66	4	3	10	10
							
*NuRD complex*							
Transcriptional repressor p66-beta	P66B_MOUSE	113	48	9	5	22	11
Methyl-CpG-binding domain protein 3	MBD3_MOUSE	77	77	11	1	38	5
							
*Transcription factors*							
Steroid hormone receptor ERR1	ERR1_MOUSE	584	492	20	15	69	55
LINE-1 type transposase domain-containing protein 1	G3UYN0_MOUSE	516	531	27	29	37	38
Upstream-binding protein 1	UBIP1_MOUSE	277	180	15	9	40	31
Zinc-finger protein 281	ZN281_MOUSE	256	61	13	5	19	8
Ras-responsive element-binding protein 1	RREB1_MOUSE	235	149	18	11	16	11
Zinc-finger protein 462	A2SW42_MOUSE	229	68	31	9	15	5
Nuclear receptor subfamily 0 group B member 1	NR0B1_MOUSE	195	86	9	7	26	23
Transcription factor CP2-like protein 1	TF2L1_MOUSE	160	165	11	8	33	35
Zinc-finger protein 687	ZN687_MOUSE	159	87	12	4	14	5
Runt-related transcription factor 1	RUNX1_MOUSE	149	46	10	6	41	19
Alpha-globin transcription factor CP2	TFCP2_MOUSE	124	68	7	3	26	14
Protein Prdm2	A2A7B5_MOUSE	105	45	8	6	7	7
Transcription factor jun-B	JUNB_MOUSE	97	29	3	1	16	7
Undifferentiated embryonic cell transcription factor 1	UTF1_MOUSE	85	76	6	6	31	37
Transcription factor EB	TFEB_MOUSE	63	60	2	4	6	9
Zinc-finger protein 592	ZN592_MOUSE	62	30	7	2	8	4
MAX gene-associated protein	MGAP_MOUSE	59	30	7	3	3	1
Zinc-finger protein 655	Q6P9P9_MOUSE	48	110	5	11	9	28
Zinc-finger protein 143	ZN143_MOUSE	32	33	2	5	7	11
