# Supplementary material for: Fgf and Esrrb integrate epigenetic and transcriptional networks that regulate self-renewal of trophoblast stem cells
Source: Nat Commun. 2015 Jul 24;6:7776. doi: 10.1038/ncomms8776 (PMC4525203; doi:10.1038/ncomms8776)
Supplement: Supplementary Information — Supplementary Figures 1-10, Supplementary Tables 1-5 and Supplementary References [file ncomms8776-s1.pdf]

Supplementary Figure 1

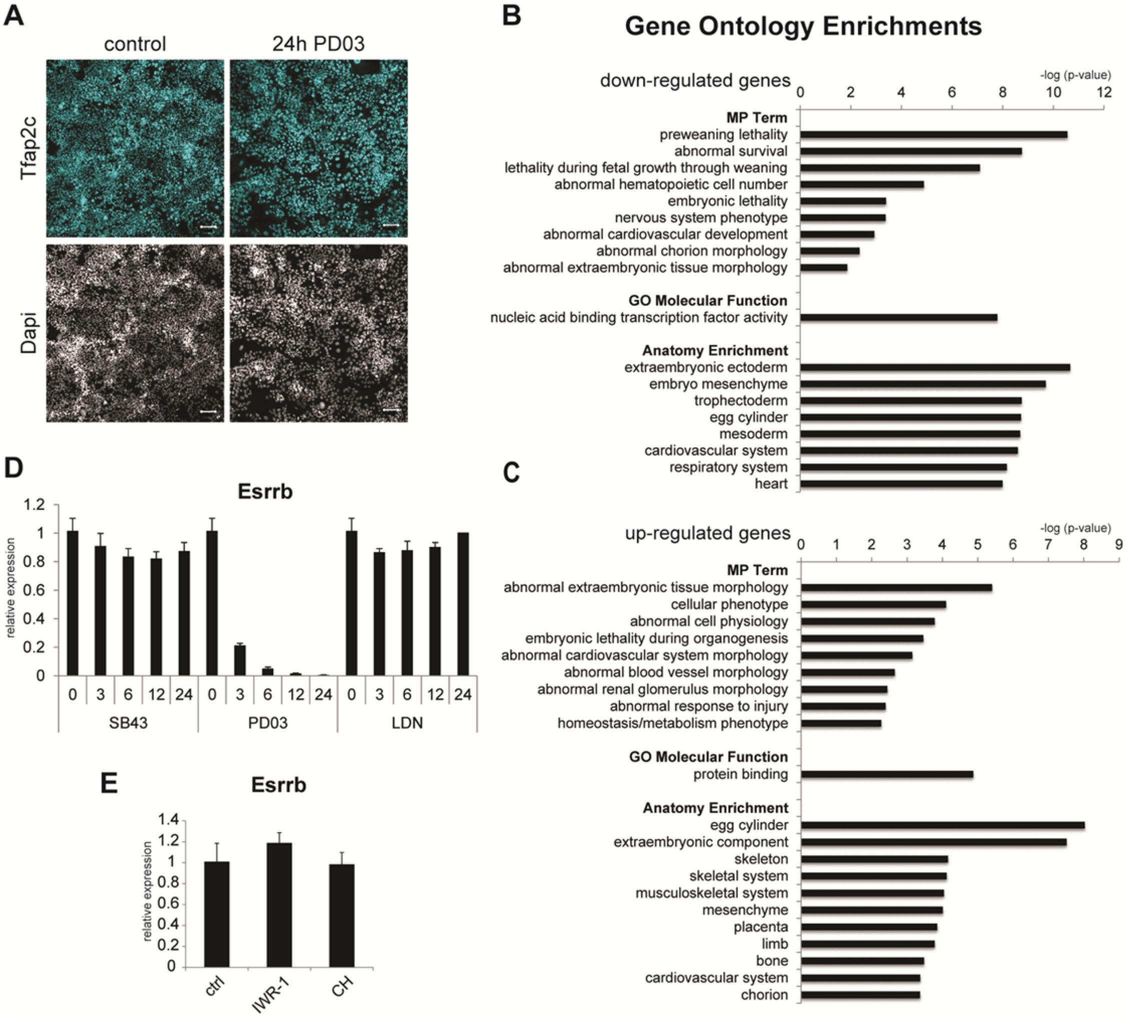

Supplementary Figure 1 Esrrb-driven regulation of TS cells.

(a) Immunostaining for Tfap2c after 24h treatment with the PD03 inhibitor. Magnification bars: 100 µm. **(b)** Gene Ontology Enrichments of genes that were down-regulated or **(c)** up-regulated upon the PD03 treatment of TS cells. **(d)** Gene expression analysis of TS cells grown for 3, 6,12, and 24h, in the presence of the inhibitors SB431542 (SB43; inhibitor of Nodal/activin), PD0325901 (PD03; Mek) or LDN (Bmp4). Bars represent three technical replicates +/- SD. **(e)** Gene expression analysis of TS cells grown for 72h in the presence of Chiron (CH; Gsk3) and IWR-1 (Wnt pathway) inhibitors. Bars represent three technical replicates +/- SD.

Supplementary Figure 2

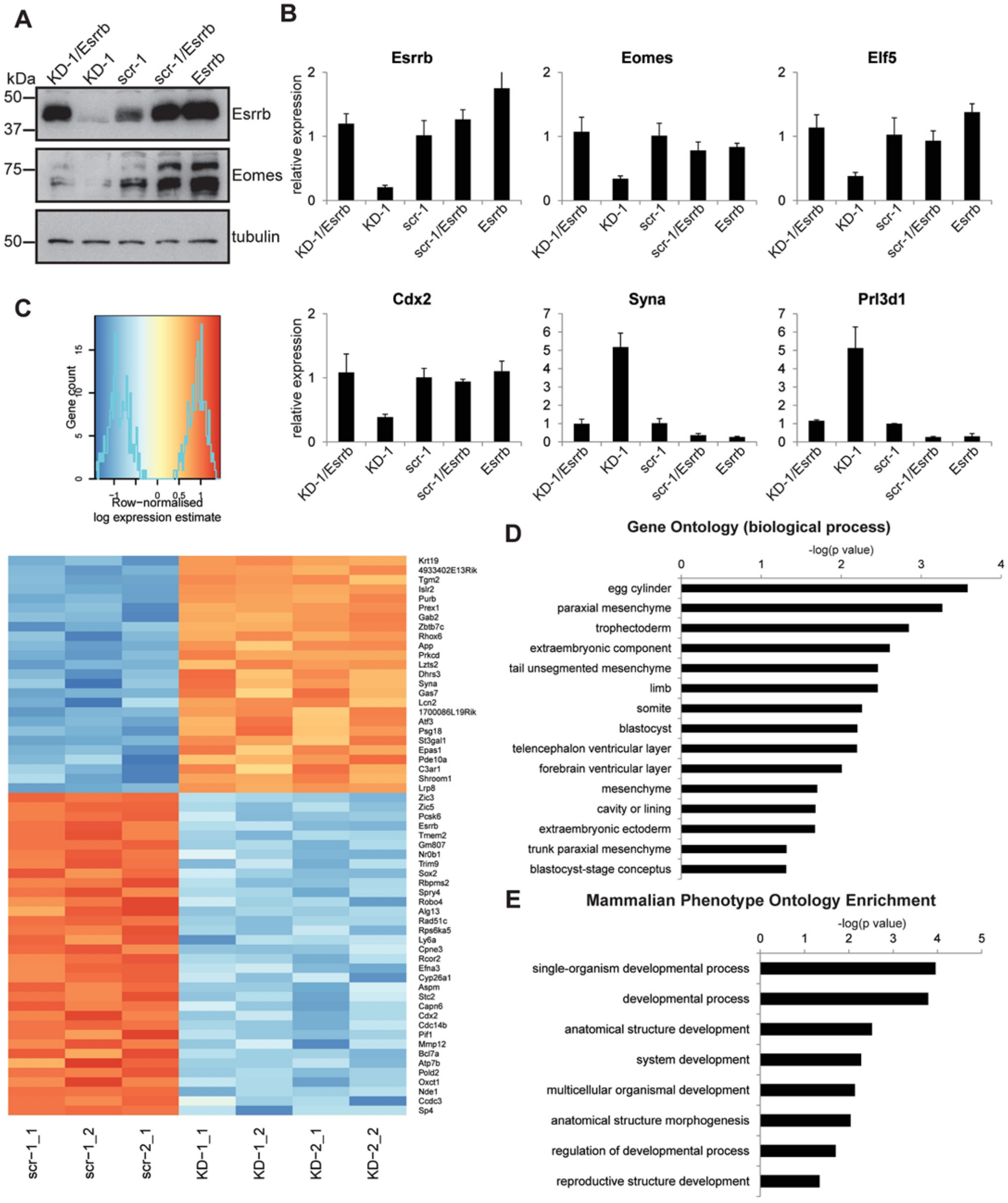

Supplementary Figure 2 *Esrrb* depletion results in TS cell differentiation.

(a) Representative Western blot showing restored expression of Esrrb and Eomes in the *Esrrb* rescue line (KD-1/Esrrb) compared to control (scr-1) and *Esrrb* overexpressing (scr-1/Esrrb; Esrrb) lines. Full WB scan can be viewed in Supplementary Fig. 10g. **(b)** Gene expression analysis of *Esrrb* knockdown TS cell line rescued with an Esrrb expression construct (KD-1/Esrrb; generated by co-transfecting KD-1 shRNA targeted against the 3'-UTR with an *Esrrb* coding region) compared to *Esrrb* knockdown (KD-1), control (scr-1) and *Esrrb* overexpressing (scr-1/Esrrb; Esrrb) lines. **(c)** Heat map of differentially expressed genes (at posterior probability > 0.9) in *Esrrb* knockdown TS cells (KD-1_1, KD-1_2, KD-2_1, KD-2_2) compared to controls (scr-1_1, scr-1_2, scr-2_1). **(d)** Gene Ontology enrichment for biological processes of genes affected by *Esrrb* depletion. **(e)** Mammalian Phenotype Ontology enrichment analysis of genes affected by *Esrrb* depletion.

Supplementary Figure 3

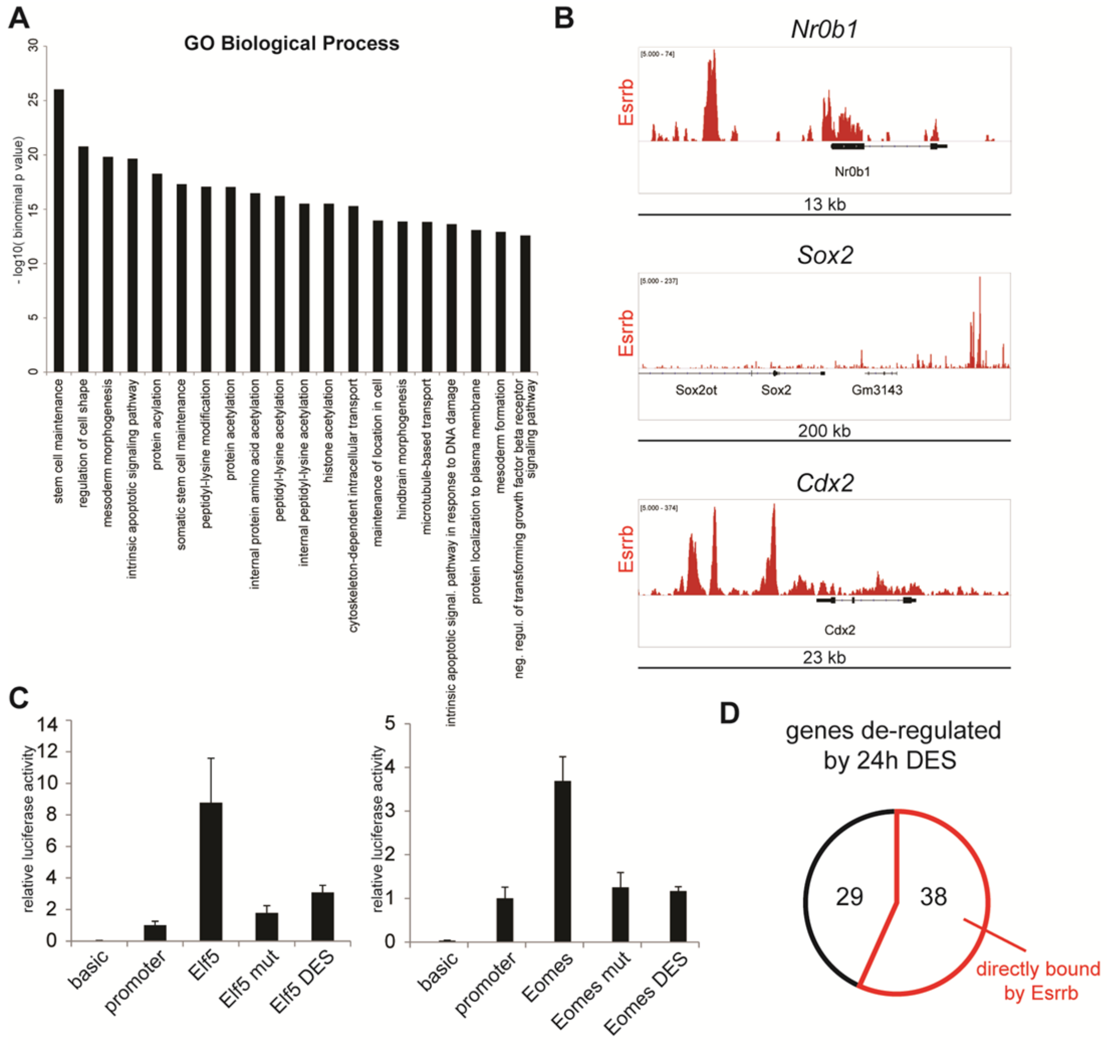

Supplementary Figure 3 Esrrb binds TS cell specific genes.

(a) 20 top terms of the GREAT ontology enrichments for ES cell specific Esrrb peaks. **(b)** Examples of Esrrb binding profiles at the *Nr0b1*, *Sox2* and *Cdx2* loci in TS cells. **(c)** (left) Luciferase reporter assays of TS cells transiently transfected with a putative wild-type (wt) *Elf5* enhancer construct (Elf5: pGL3-promoter-Elf5), with a construct containing mutated Esrrb binding sites (Elf5 mut), with the wt *Elf5* enhancer construct in the presence of DES inhibitor (Elf5 DES) and controls: (basic: pGL3-basic and promoter: pGL3-promoter); (right) Luciferase reporter assays of TS cells transiently transfected with a putative wt *Eomes* enhancer construct (Eomes: pGL3-promoter-Eomes), with a construct containing mutated Esrrb binding sites (Eomes mut), with the wt *Eomes* enhancer construct in the presence of DES inhibitor (Eomes DES) and controls: (basic: pGL3-basic and promoter: pGL3-promoter). **(d)** Pie chart depicting the proportion of genes differentially expressed upon 24h DES treatment (RNA-seq, at posterior proximity score > 0.8) that are also bound by Esrrb as identified by ChIP-seq analysis.

Supplementary Figure 4

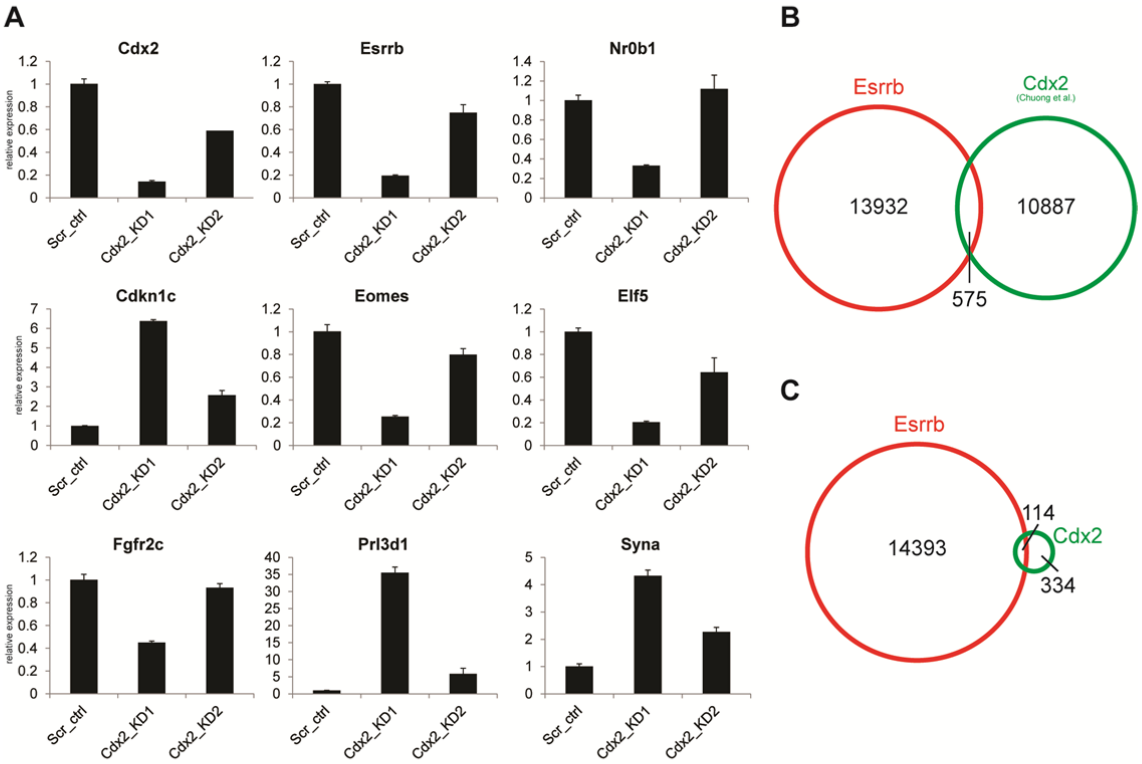

Supplementary Figure 4 *Cdx2* depletion results in TS cell differentiation.

(a) RT-QPCR analysis of Cdx2 knockdown (Cdx2_KD-1, Cdx2_ KD-2) and control (Scr_ctrl) TS cells 5 days after transfection. TS cell markers (*Esrrb*, *Elf5*, *Eomes, Fgfr2c* and *Nr0b1*) were down-regulated in *Cdx2* depleted cells whereas differentiation markers (*Syna*, *Cdkn1c* and *Prl3d1*) were up-regulated. Bars indicate the mean of at least three biological replicates +/- S.E.M. **(b)** Venn diagram showing overlap between ChIP-seq peaks for Esrrb (this study) and Cdx2 (peaks as published by Chuong et al.[1]). **(c)** Venn diagram showing overlap between Esrrb and Cdx2 ChIP-seq peaks. Here, Cdx2 peaks were called by us using the raw data published by Chuong et al.[1] and applying identical analysis criteria as for Esrrb.

Supplementary Figure 5

A

Protein ID	Accession	Score	Coverage	# Unique Peptides	Description
CDX2	P43241	552.99	44.05	13	Homeobox protein CDX-2
TBA1C	P68373	568.27	44.32	14	Tubulin alpha-1C chain
DDX3L	P16381	401.26	20.15	11	Putative ATP-dependent RNA helicase Pl10
H2B2B	Q64525	336.42	49.21	7	Histone H2B type 2-B
FBRL	P35550	91.36	17.13	3	rRNA 2'-O-methyltransferase fibrillarin
HMGB1	P63158	64.17	22.33	3	High mobility group protein B1
H2A1H	Q8CGP6	338.92	58.59	2	Histone H2A type 1-H
PSA7	Q9Z2U0	59.77	16.53	2	Proteasome subunit alpha type-7
OLA1	Q9CZ30	58.55	7.58	2	Obg-like ATPase 1
RBM3	O89086	45.31	20.92	2	Putative RNA-binding protein 3

B

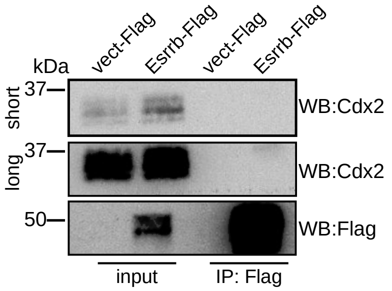

D

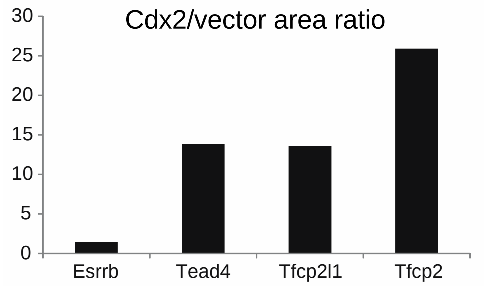

C

Protein	Accession Number	score	unique peptides	coverage
**Homeobox protein CDX-2**	**CDX2_MOUSE**	**208**	**5**	**14%**
Bromodomain PHD finger transcription factor	A2A654_MOUSE	231	12	5%
Upstream-binding protein 1	UBIP1_MOUSE	204	6	16%
Heterogeneous nuclear ribonucleoprotein U-like protein 2	HNRL2_MOUSE	141	5	9%
Transcription factor CP2-like protein 1	TF2L1_MOUSE	136	4	14%
Bifunctional polynucleotide phosphatase/kinase	PNKP_MOUSE	126	4	15%
X-ray repair cross-complementing protein 6	XRCC6_MOUSE	124	6	14%
DNA polymerase beta	DPOLB_MOUSE	96	3	15%
Histone H3.2	H32_MOUSE	92	2	29%
Protein BC018507	E9Q286_MOUSE	88	4	3%
Tyrosine-protein phosphatase non-receptor type 14	PTN14_MOUSE	83	2	3%
X-ray repair cross-complementing protein 5	XRCC5_MOUSE	77	3	5%
A-kinase anchor protein 8-like	AKP8L_MOUSE	72	2	4%
Origin recognition complex subunit 4	ORC4_MOUSE	64	2	8%
Transcriptional enhancer factor TEF-3	TEAD4_MOUSE	64	6	15%
DBIRD complex subunit ZNF326	ZN326_MOUSE	61	2	5%
U3 small nucleolar RNA-associated protein 14 homolog A	UT14A_MOUSE	59	3	13%
DNA repair protein XRCC1	XRCC1_MOUSE	57	3	7%
RNA-binding protein 7	RBM7_MOUSE	57	4	19%
Transcriptional regulator ATRX	ATRX_MOUSE	56	3	1%
Protein Kif23	E9Q5G3_MOUSE	55	2	2%
Eomesodermin homolog	EOMES_MOUSE	54	3	7%
THO complex subunit 5 homolog	THOC5_MOUSE	54	2	5%
Histone-lysine N-methyltransferase EHMT1	EHMT1_MOUSE	53	2	2%
Grainyhead-like protein 2 homolog	GRHL2_MOUSE	51	3	8%
Zinc finger CCHC domain-containing protein 8	ZCHC8_MOUSE	51	3	7%
Pre-rRNA-processing protein TSR1 homolog	TSR1_MOUSE	48	2	3%
DNA mismatch repair protein Msh6	MSH6_MOUSE	47	3	3%
Cleavage and polyadenylation specificity factor subunit 7	CPSF7_MOUSE	46	2	4%
Krueppel-like factor 5	KLF5_MOUSE	45	2	9%
Pinin	PININ_MOUSE	45	3	5%
PHD finger protein 10	PHF10_MOUSE	44	2	12%
THO complex subunit 1	THOC1_MOUSE	41	2	4%
Zinc finger protein 592	ZN592_MOUSE	41	2	3%
CDKN2A-interacting protein	CARF_MOUSE	40	2	5%
Exosome complex component RRP45	EXOS9_MOUSE	40	3	8%
DNA replication licensing factor MCM4	MCM4_MOUSE	39	2	3%
Host cell factor 1	HCFC1_MOUSE	36	2	2%
MAP7 domain-containing protein 1	MA7D1_MOUSE	35	2	3%
p21-activated protein kinase-interacting protein 1	PK1IP_MOUSE	30	2	9%
DNA-directed RNA polymerase I subunit RPA2	RPA2_MOUSE	29	2	3%
Splicing factor 3A subunit 2	G3UVU2_MOUSE	29	2	5%
Histone-lysine N-methyltransferase SETD2	SETD2_MOUSE	27	2	1%

Supplementary Figure 5 Cdx2 and Esrrb function independently to maintain the stem cell state of TS cells.

(a) RIME (rapid immunoprecipitation mass spectrometry of endogenous proteins) analysis of Cdx2-interacting proteins: the table represents proteins identified with at least 2 unique peptides in the Cdx2 sample and none in the IgG control. **(b)** Representative Esrrb-3xFlag immunoprecipitates analysed by Western blot probed with anti-Cdx2 (Cdx2) and anti-Flag (Flag) antibodies showing the lack of interaction between Esrrb and Cdx2 transcription factors. Full WB scan can be viewed in Supplementary Fig. 10h. **(c)** Cdx2-interacting proteins as identified by mass spectrometry analysis using tagged Cdx2-3xFlag and vector control TS lines. High-confidence hits are shown. **(d)** As the mass spectrometry identified a small number of Esrrb peptides in both Cdx2-3xFlag and vector control immunoprecipitates, we quantified the peptide spectra. The chart depicts enrichment (area) ratios of peptides identified in Cdx2-3xFlag and vector immunoprecipitates for Esrrb and other proteins that serve as positive controls.

Supplementary Figure 6

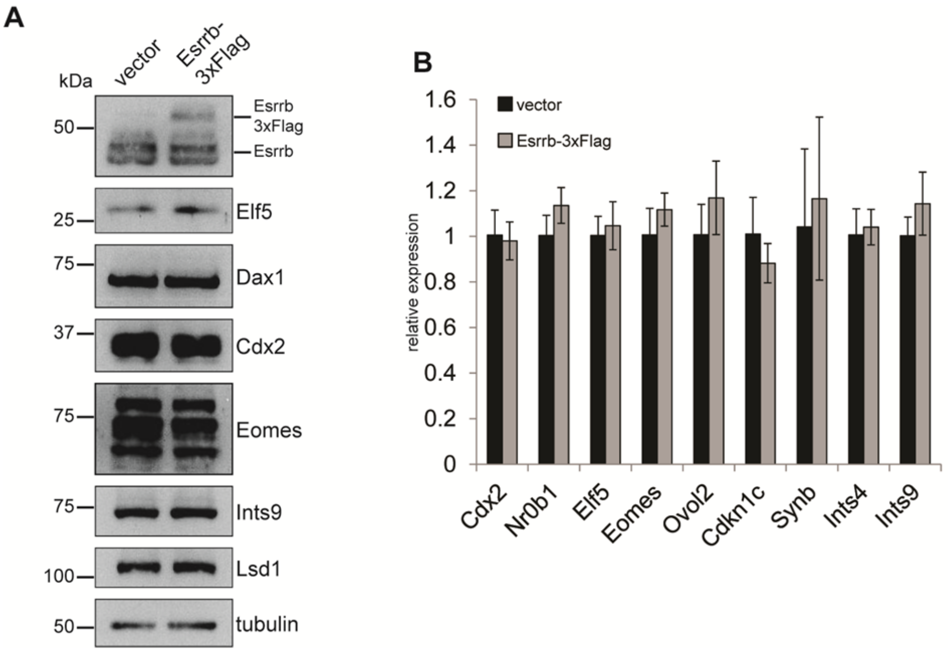

Supplementary Figure 6 Forced expression of Esrrb-3xFlag does not perturb the TS cell state.

(a) Representative Western blot analysis of proteins expressed in the Esrrb-3xFlag overexpressing TS cell line in comparison to vector control TS cells. Full WB scan can be viewed in Supplementary Fig. 10i. **(b)** RT-QPCR gene expression analysis of Esrrb-3xFlag overexpressing line compared to vector control. Bars represent the mean of two biological and six technical replicates +/- SD.

Supplementary Figure 7

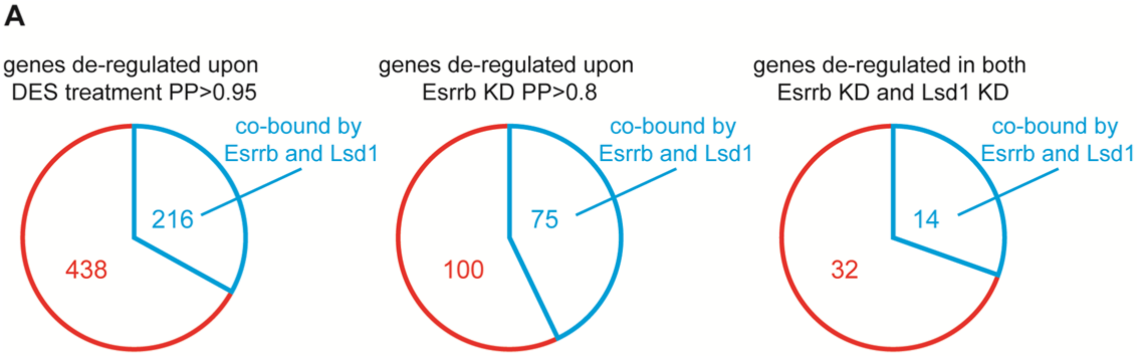

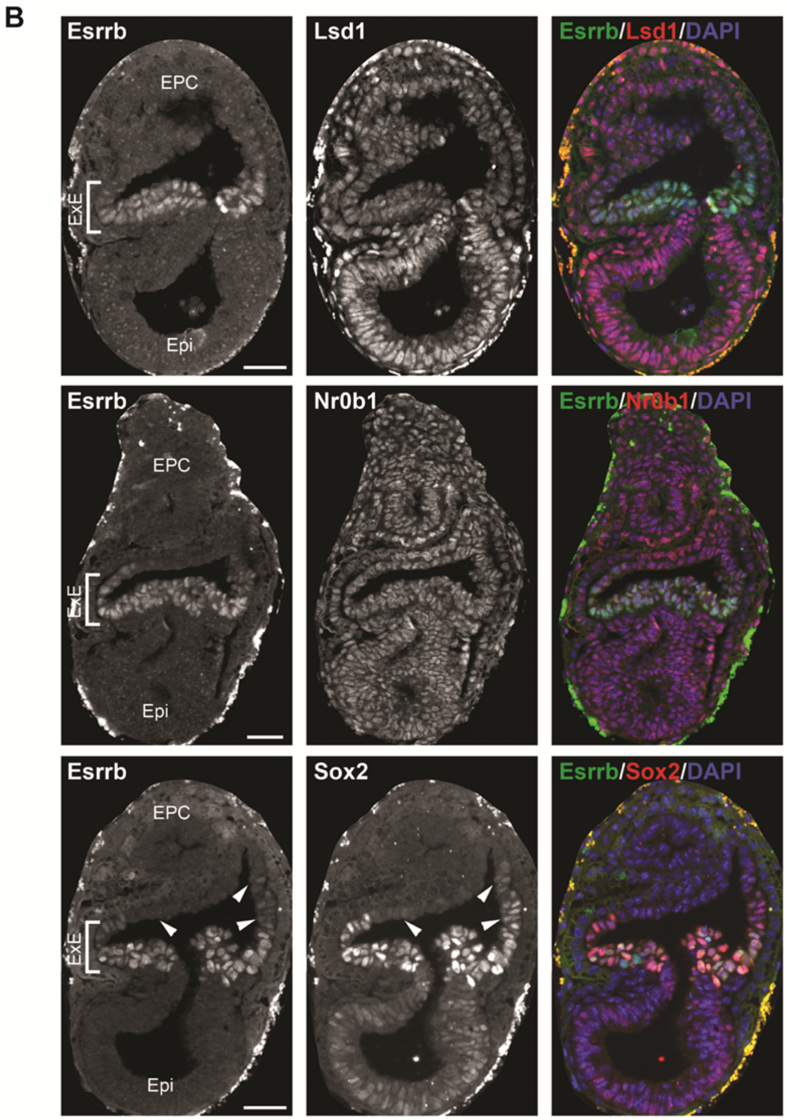

Supplementary Figure 7 Expression of Esrrb, Nr0b1, Lsd1 and Sox2 in E6.5 mouse conceptuses.

(a) Pie charts depicting the proportion of genes differentially expressed upon DES treatment (left; RNA-seq, at posterior proximity score > 0.95) that are also co-bound by Esrrb and Lsd1 as identified by ChIP-seq analysis. (middle) Pie chart showing the proportion of genes differentially expressed upon Esrrb KD (RNA-seq, at posterior proximity score > 0.8) that are also bound by Esrrb and Lsd1. (right) Pie chart indicating the proportion of genes differentially expressed upon both Esrrb KD and Lsd1 KD that are also co-bound by Esrrb and Lsd1 as identified by ChIP-seq analysis. **(b)** Double-immunofluorescence stainings of paraffin sections of E6.5 conceptuses for Esrrb combined with Lsd1 (upper row), Nr0b1 (middle row) and Sox2 (bottom row). Photographs are from 7µm sections of the same conceptus at different section planes; the informative extraembryonic ectoderm (ExE) region is highlighted for each section. Note widespread expression of Lsd1 and Nr0b1 in both embryonic and extraembryonic compartments in contrast to the narrow expression domain of Esrrb that is confined to the ExE only. Of note is also the slightly wider expression domain of Sox2 that persists in trophoblast cells farther away from the epiblast (Epi), which provides the source of Fgf4, towards the ectoplacental cone (EPC) than Esrrb. This finding corroborates the in vitro observation that Esrrb is the fastest responder to Fgf/Mek inhibition (Fig. 1). Magnification bars: 50 µm.

Supplementary Figure 8

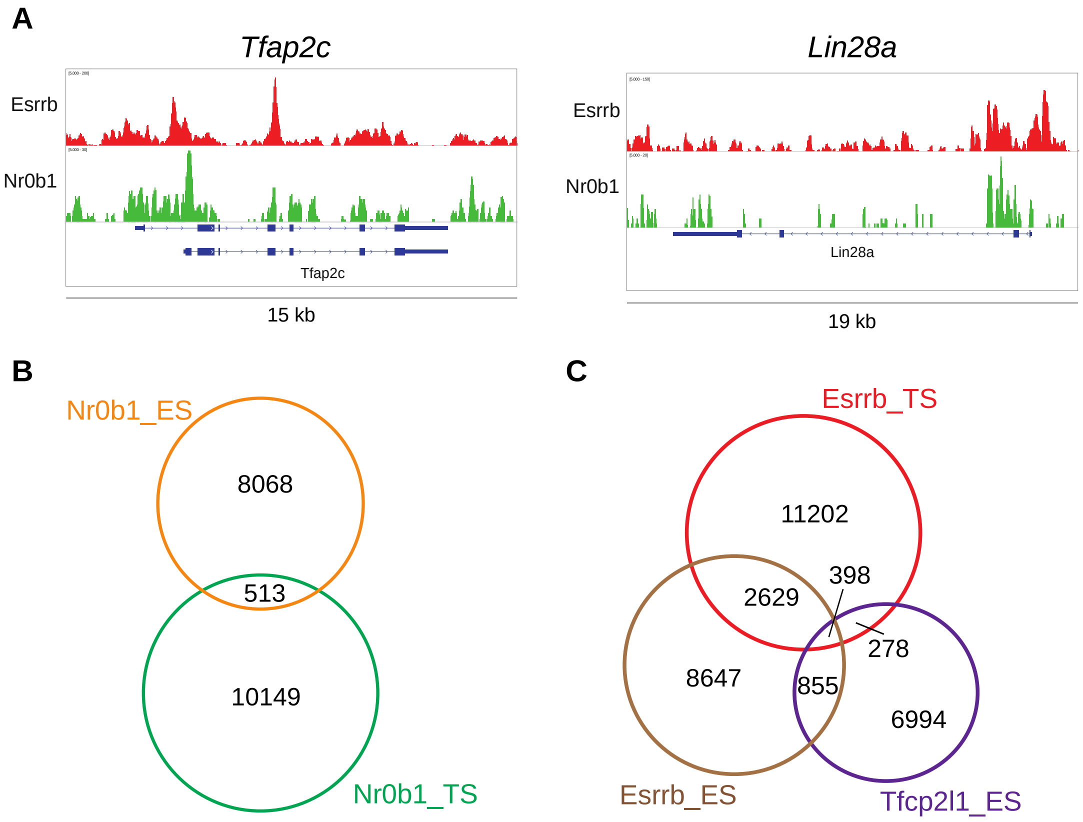

Supplementary Figure 8 Esrrb interacts with Nr0b1 and shares gene targets.

(a) Examples of Esrrb and Nr0b1 binding profiles at the *Tfap2c* and *Lin28a* loci. **(b)** A Venn diagram showing the number of the Nr0b1 TS cell specific peaks, ES cell specific peaks and those overlapping in both types of stem cell; Nr0b1 ES ChIP-seq are from Kim et al.[2]. **(c)** A Venn diagram showing the number of overlapping peaks between Esrrb in TS cells (Esrrb_TS), Esrrb in ES cells (Esrrb_ES; Chen et al.[3]) and Tfcp2l1 in ES cells (Tcfcp2l1_ES; Chen et al.[3]).

Supplementary Figure 9

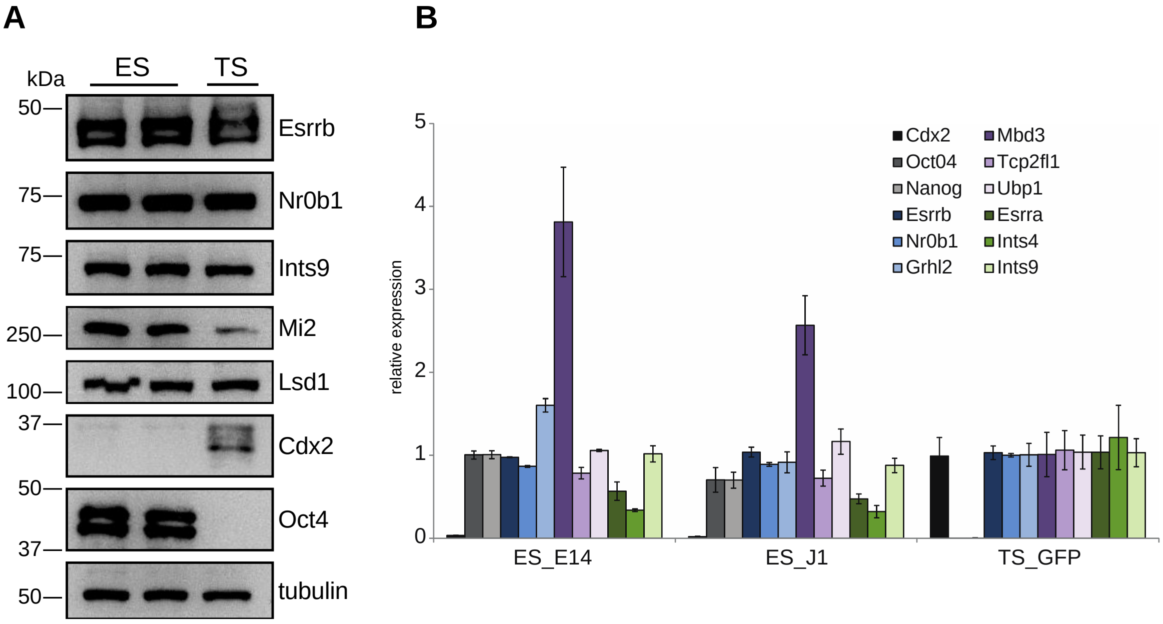

Supplementary Figure 9 TS and ES cells express similar levels of Esrrb, Nr0b1 and Lsd1. **(a)** Representative Western blot showing protein levels of Esrrb, Nr0b1, Ints9, Mi2beta and Lsd1 in ES cells and TS cells. Oct4 (expressed only in ES cells), Cdx2 (expressed only in TS cells) and tubulin serve as controls. Full WB scans can be viewed in Supplementary Fig. 10j. **(b)** RT-QPCR analysis comparing expression levels of chosen genes in ES and TS cells. Bars represent biological triplicates +/- S.E.M.

Supplementary Figure 10 Full Western blot scans.

A

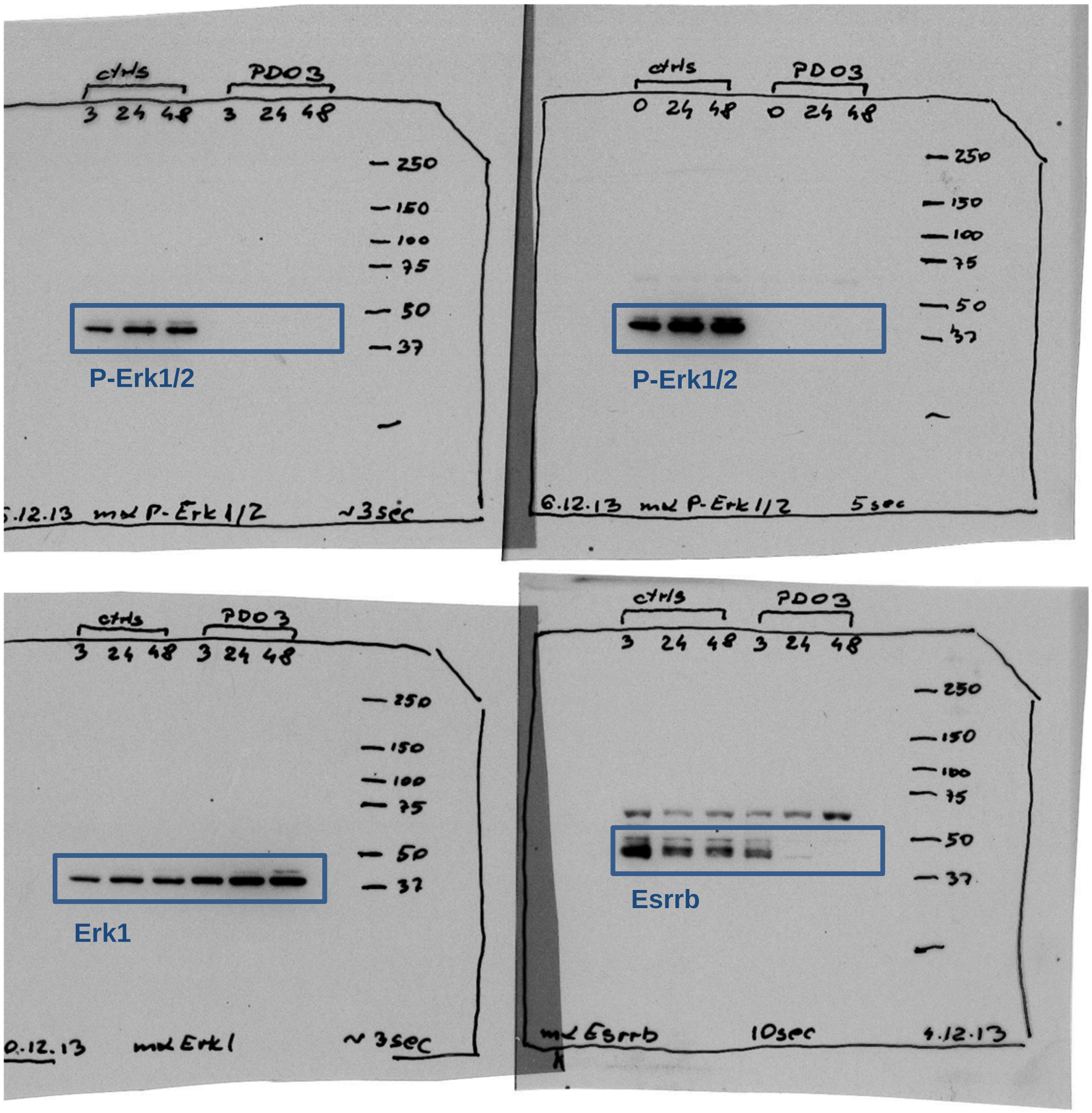

B

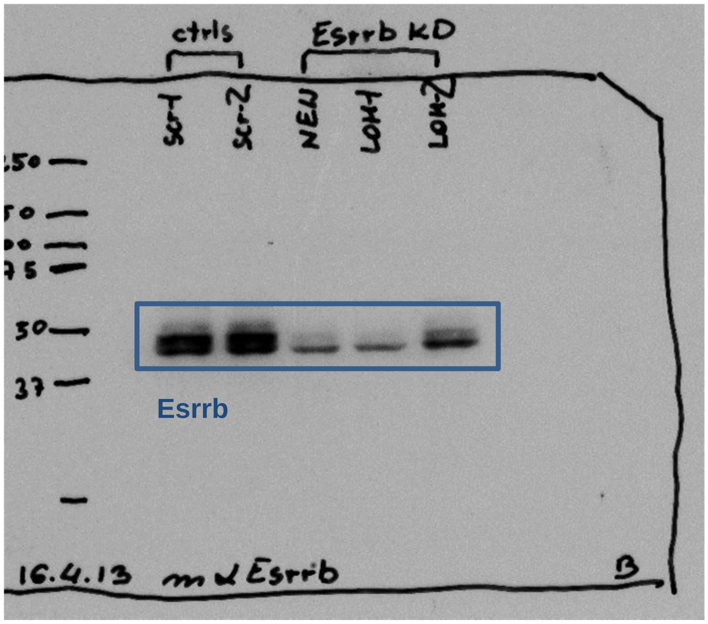
ctrls
Esrrb KD
Scr-1
Scr-2
NEW
LOH-1
LOH-2
50
37
Esrrb
16.4.13

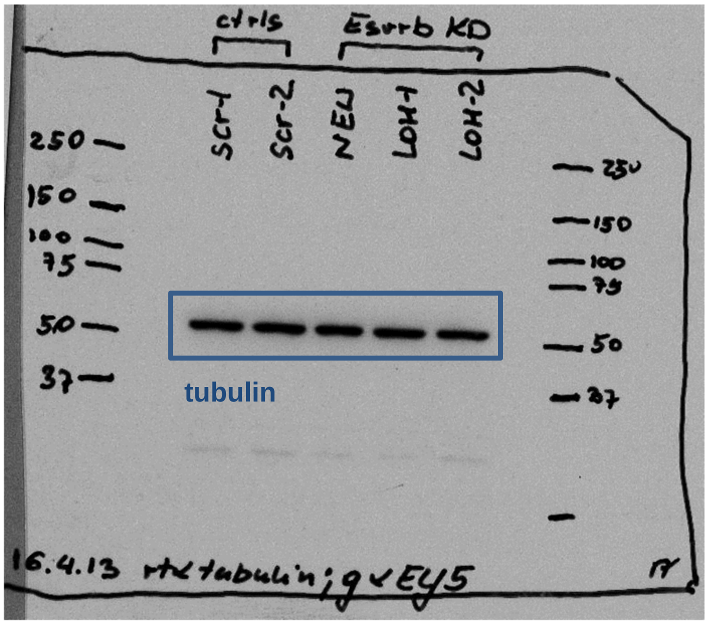
ctrls
Esrrb KD
Scr-1
Scr-2
NEW
LOH-1
LOH-2
250
150
100
75
50
37
tubulin
16.4.13

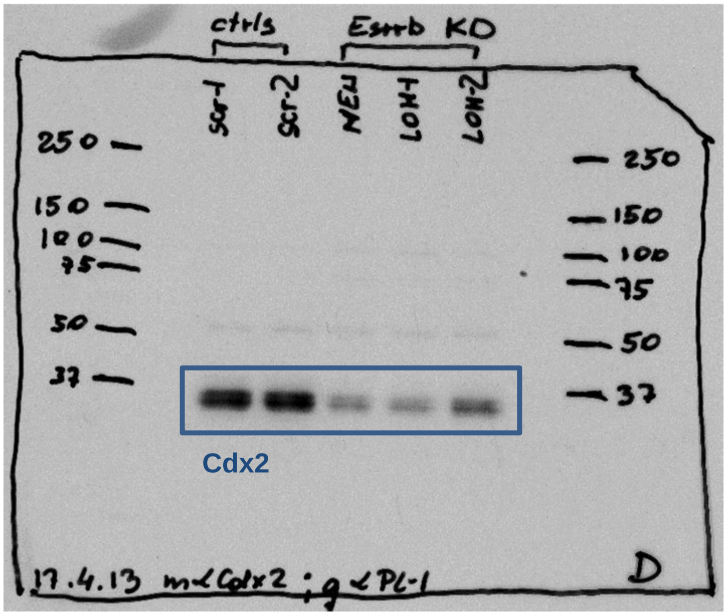
ctrls
Esrrb KD
Scr-1
Scr-2
NEW
LOW-1
LOW-2
250
150
100
75
50
37
Cdx2
D
17.4.13 mαCdx2 ; gαPL-1

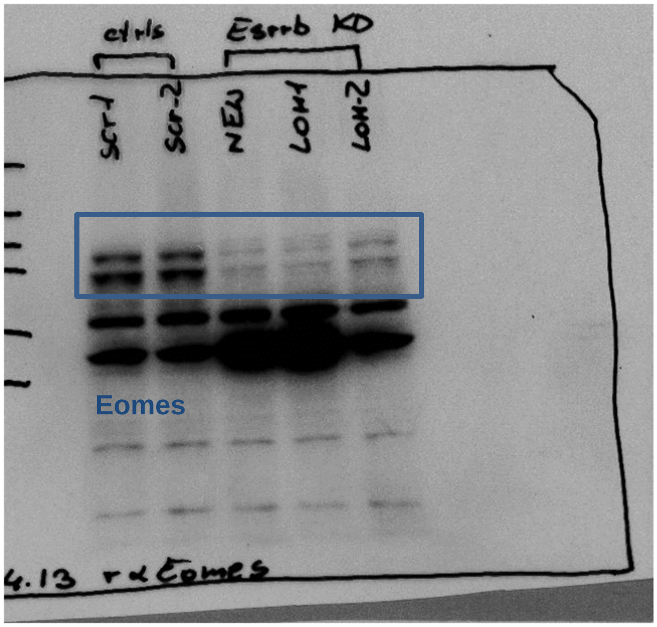
ctrls
Esrrb KD
Scr-1
Scr-2
NEW
LOW-1
LOW-2
Eomes
4.13 rαEomes

C

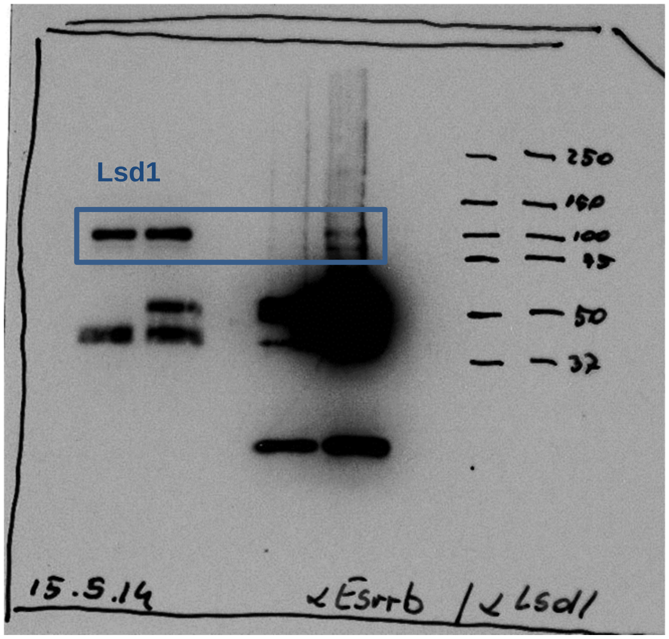

D

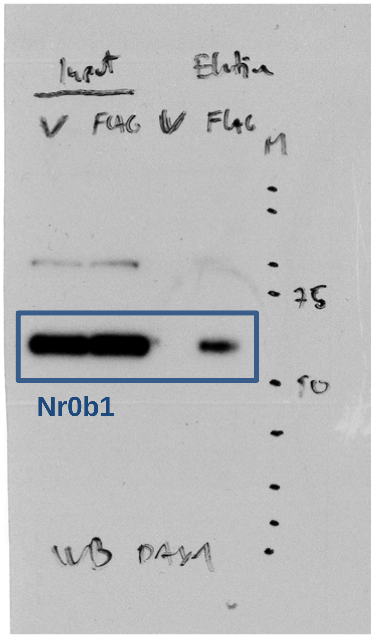

E

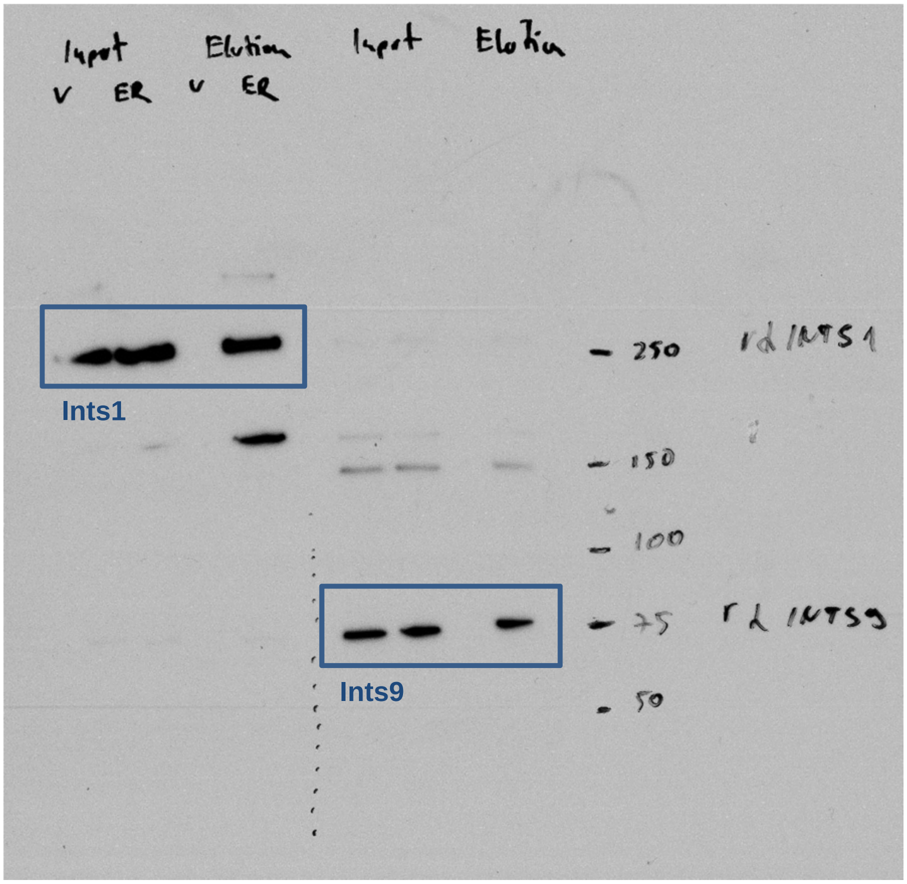
Input
Elution
V
ER
V
ER
Input
Elution
Ints1
250
150
100
75
50
Ints9

F

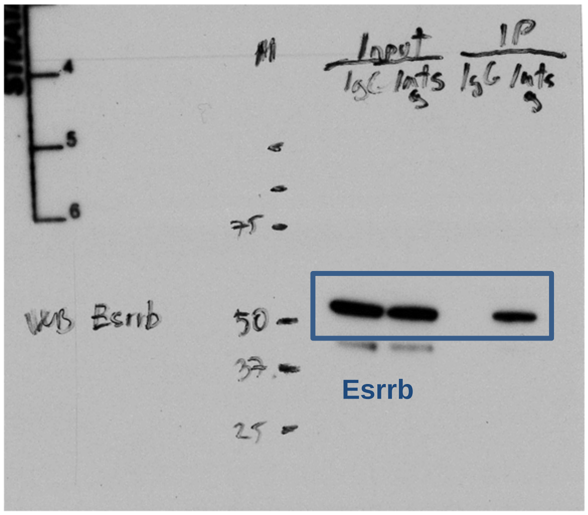
M
Input
IP
IgG
Ints9
IgG
Ints9
75
50
37
25
WB Esrrb
Esrrb

G

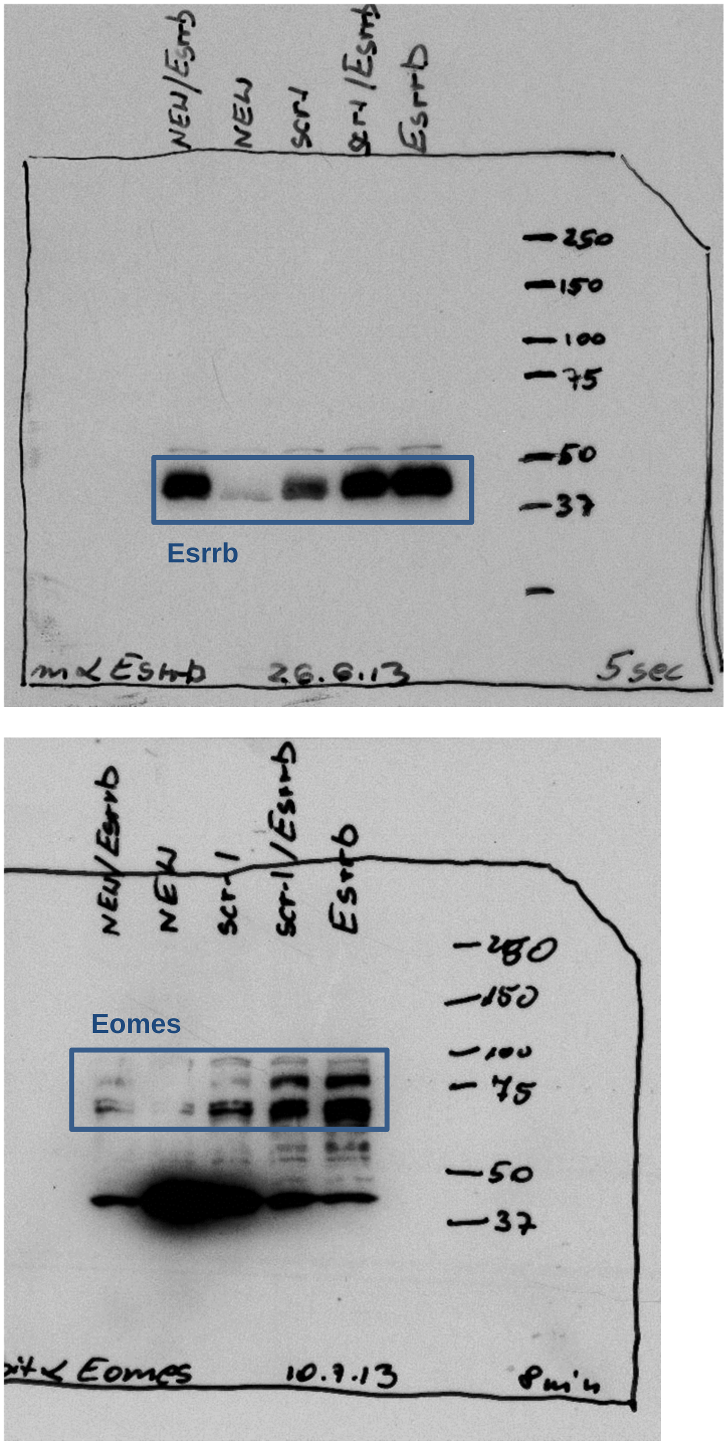
NEW/Esrrb
NEW
scr-1
scr-1/Esrrb
Esrrb
250
150
100
75
50
37
Esrrb
26.6.13
5 sec
Eomes
250
150
100
75
50
37
Eomes
10.7.13
8 min

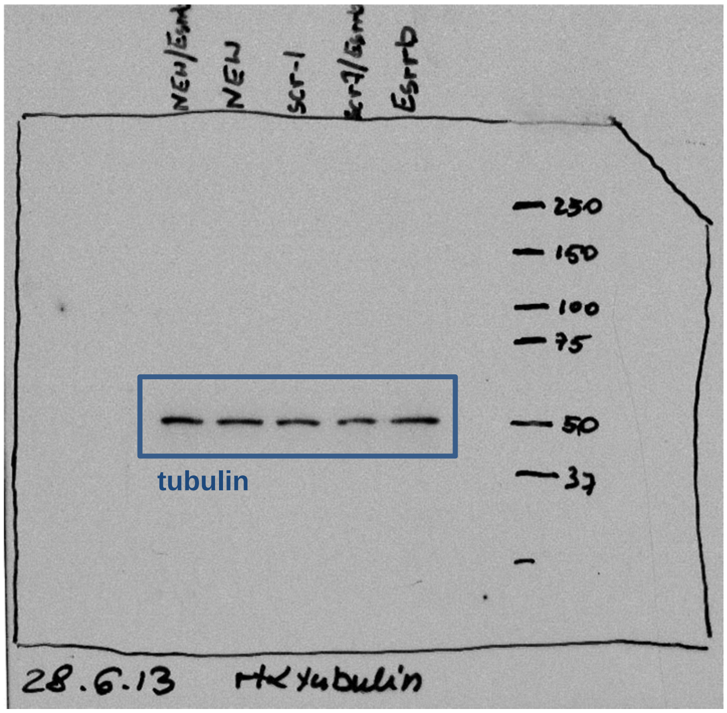
NEW/Esrrb
NEW
scr-1
scr-1/Esrrb
Esrrb
250
150
100
75
50
37
tubulin
28.6.13
tubulin

H

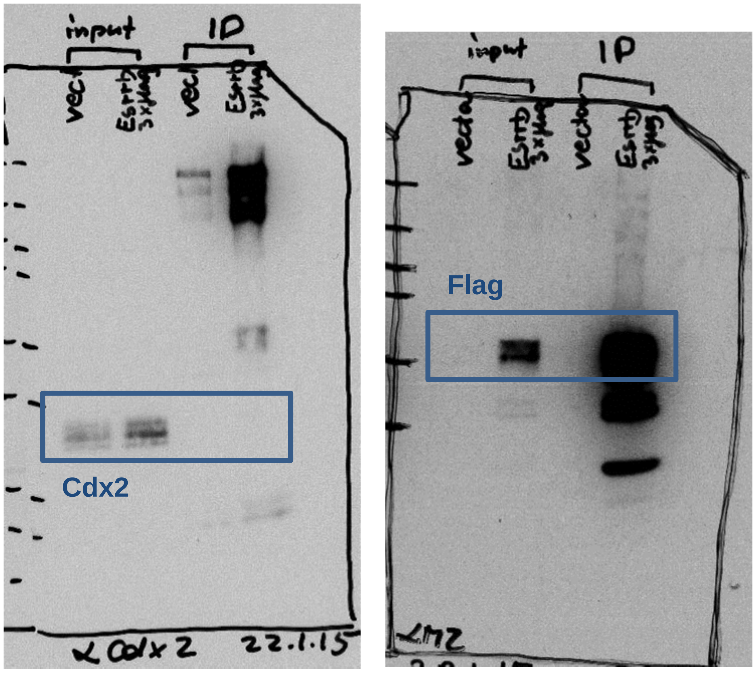
input
IP
vec
Esrrb 3xflag
vec
Esrrb 3xflag
Cdx2
Cdx2
22.1.15
input
IP
vector
Esrrb 3xflag
vector
Esrrb 3xflag
Flag

I

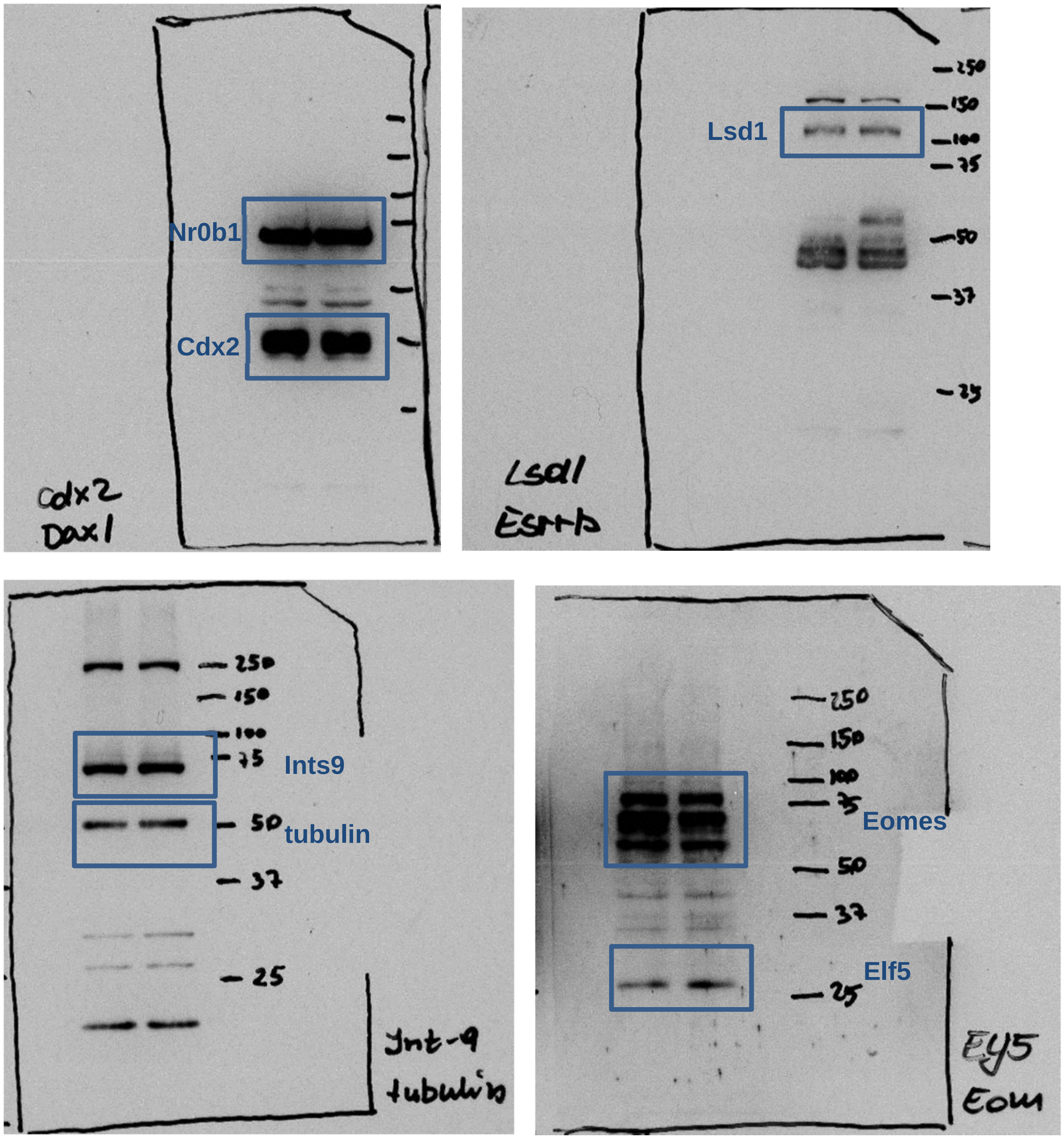
Nr0b1
Cdx2
Cdx2
Dax1
Lsd1
250
150
100
75
50
37
25
Lsd1
Esrrb
250
150
100
75
Ints9
50
tubulin
37
25
Int-9
tubulin
250
150
100
75
Eomes
50
37
Elf5
25
Elf5
Eom

J

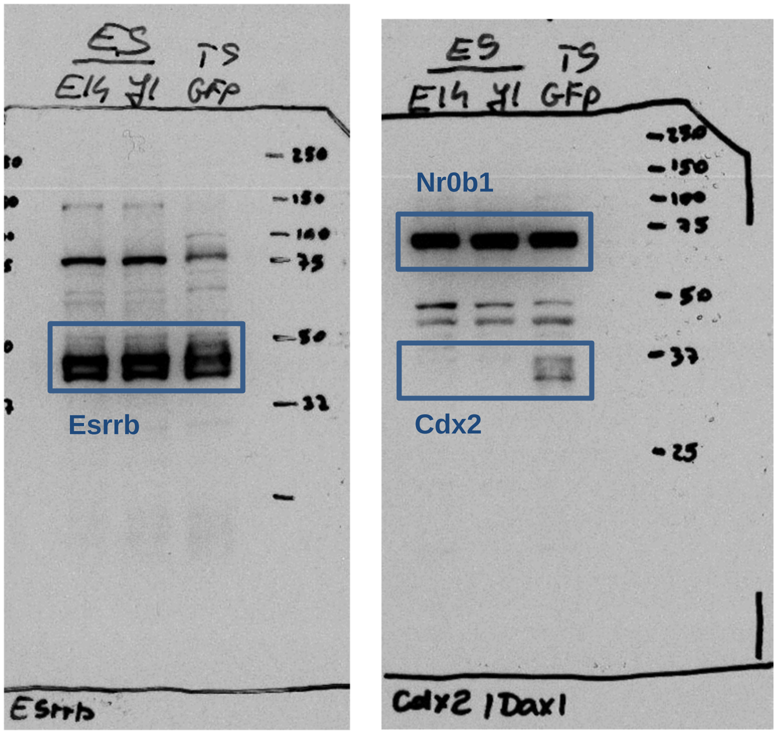
ES
TS
E14
Y1
GFP
250
150
100
75
50
37
Esrrb
Esrrb
ES
TS
E14
Y1
GFP
250
150
100
75
50
37
25
Nr0b1
Cdx2
Cdx2 / Dax1

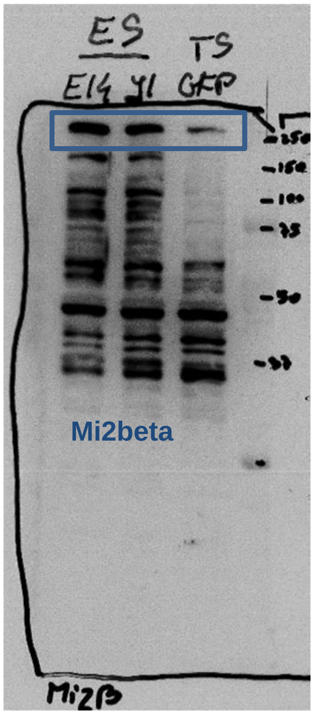
ES
TS
E14 Y1 GFP
Mi2beta
Mi2β

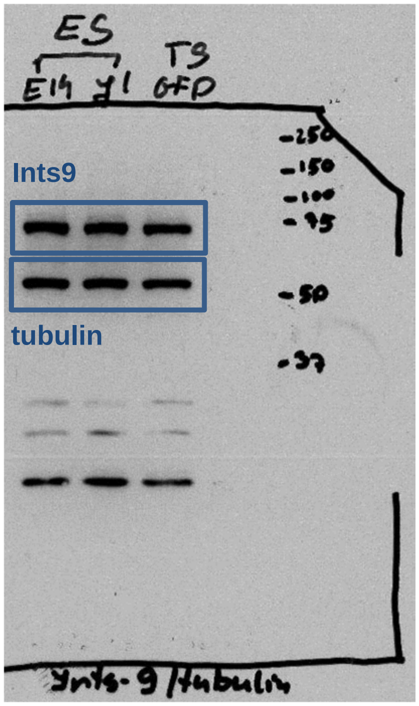
ES
TS
E14 Y1 GFP
Ints9
tubulin
-250
-150
-100
-75
-50
-37

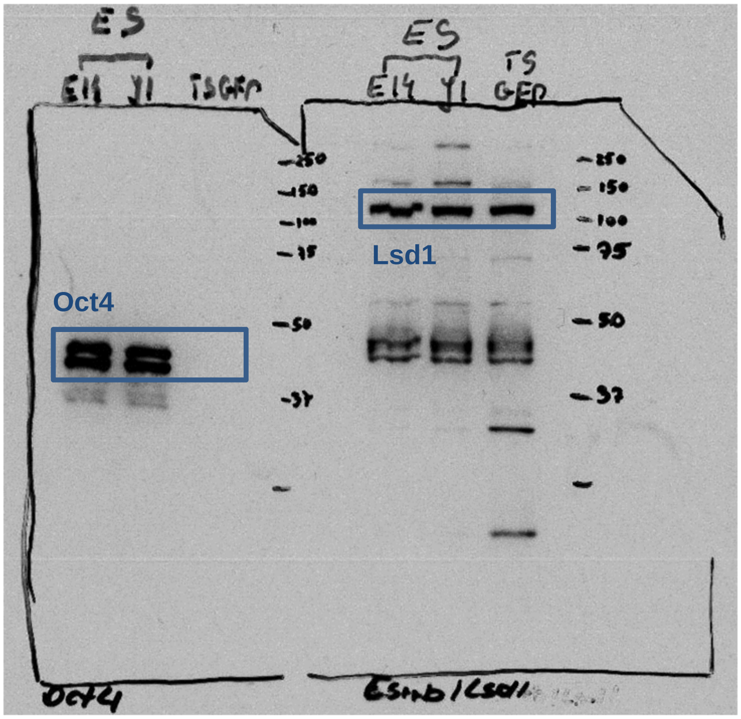
ES
E14 Y1 TSGFP
TS
E14 Y1 GFP
Oct4
Lsd1
-250
-150
-100
-75
-50
-37

Supplementary Figure 10 Primary scans of Western blots.

(a) Primary scans of Western blots as in Figure 1a. **(b)** Primary scans of Western blots as in Figure 2f. **(c)** Primary scans of Western blots as in Figure 4b. **(d)** Primary scans of Western blots as in Figure 5a. **(e)** Primary scans of Western blots as in Figure 5e. **(f)** Primary scans of Western blots as in Figure 5f. **(g)** Primary scans of Western blots as in Supplementary Figure 2a. **(h)** Primary scans of Western blots as in Supplementary Figure 5b. **(i)** Primary scans of Western blots as in Supplementary Figure 6a. **(j)** Primary scans of Western blots as in Supplementary Figure 9a.

Supplementary Table 1 Lsd1-interacting proteins.

Proteins shown were identified by RIME (rapid immunoprecipitation mass spectrometry of endogenous proteins) analysis with at least 3 unique peptides in the Lsd1 sample and were not found in the IgG control

Protein ID	Accession	Score	Coverage	# Unique Peptides	Description
**KDM1A**	**Q6ZQ88**	**5267.52**	**62.13**	**42**	**Lysine-specific histone demethylase 1A**
RCOR1	Q8CFE3	1313.27	47.59	19	REST corepressor 1
GSE1	Q3U3C9	1068.22	38.75	32	Genetic suppressor element 1
RREB1	Q3UH06	693.79	22.88	24	Ras-responsive element-binding protein 1
ZN516	Q7TSH3	665.11	31.03	32	Zinc finger protein 516
SMBT2	Q5DTW2	609.81	21.22	19	Scm-like with four MBT domains protein 2
RCOR2	Q8C796	592.37	38.24	13	REST corepressor 2
HDAC1	O09106	435.70	32.57	9	Histone deacetylase 1
ZMYM2	Q9CU65	339.09	21.15	21	Zinc finger MYM-type protein 2
RCOR3	Q6PGA0	331.69	26.39	6	REST corepressor 3
HDAC2	P70288	267.60	23.77	4	Histone deacetylase 2
SPT5H	O55201	211.00	14.33	10	Transcription elongation factor SPT5
TRI14	Q8BVW3	189.49	31.82	11	Tripartite motif-containing protein 14
XPO5	Q924C1	177.45	4.32	4	Exportin-5
ADNP	Q9Z103	177.06	10.51	7	Activity-dependent neuroprotector homeobox protein
CTBP2	P56546	150.32	20.00	6	C-terminal-binding protein 2
RING2	Q9CQJ4	146.49	27.08	7	E3 ubiquitin-protein ligase RING2
CTBP1	O88712	141.53	15.87	4	C-terminal-binding protein 1
RPB1	P08775	134.06	4.97	6	DNA-directed RNA polymerase II subunit RPB1
KDM5A	Q3UXZ9	132.67	6.98	9	Lysine-specific demethylase 5A
COPE	O89079	130.98	22.08	4	Coatomer subunit epsilon
RO52	Q62191	128.99	11.70	6	E3 ubiquitin-protein ligase TRIM21
SPCS	Q6P6M7	126.26	10.52	5	O-phosphoseryl-tRNA(Sec) selenium transferase
SP16H	Q920B9	124.96	3.06	3	FACT complex subunit SPT16
HM20B	Q9Z104	124.41	28.08	8	SWI/SNF-related matrix-associated actin-dependent regulator of chromatin
PDS5A	Q6A026	121.01	3.98	4	Sister chromatid cohesion protein PDS5 homolog A
HM20A	Q9DC33	118.22	23.12	7	High mobility group protein 20A
DHX15	O35286	116.45	6.04	4	Putative pre-mRNA-splicing factor ATP-dependent RNA helicase DHX15
**ERR2**	**Q61539**	**110.62**	**12.01**	**5**	**Steroid hormone receptor ERR2**
MSH6	P54276	100.12	4.86	6	DNA mismatch repair protein Msh6
PF21A	Q6ZPK0	92.00	13.35	6	PHD finger protein 21A
MCM2	P97310	89.53	4.31	3	DNA replication licensing factor MCM2
HMGB2	P30681	83.68	12.86	3	High mobility group protein B2
CHD4	Q6PDQ2	79.88	5.85	8	Chromodomain-helicase-DNA-binding protein 4
MTA3	Q924K8	79.30	8.80	4	Metastasis-associated protein MTA3
UBP7	Q6A4J8	77.14	6.71	5	Ubiquitin carboxyl-terminal hydrolase 7
PHC2	Q9QWH1	68.20	5.29	4	Polyhomeotic-like protein 2
RFA1	Q8VEE4	53.28	6.58	3	Replication protein A 70 kDa DNA-binding subunit
LKHA4	P24527	51.85	7.04	3	Leukotriene A-4 hydrolase
PSPC1	Q8R326	50.90	8.03	3	Paraspeckle component 1
FOLC	P48760	38.99	7.33	3	Folylpolyglutamate synthase, mitochondrial
AP2C	Q61312	38.86	12.69	4	Transcription factor AP-2 gamma
DNJA1	P63037	34.72	9.82	3	DnaJ homolog subfamily A member 1
EMAL2	Q7TNG5	33.88	9.09	4	Echinoderm microtubule-associated protein-like 2
S23IP	Q6NZC7	33.39	3.51	3	SEC23-interacting protein
RPB2	Q8CFI7	31.66	3.15	3	DNA-directed RNA polymerase II subunit RPB2
SUGT1	Q9CX34	29.59	11.61	4	Suppressor of G2 allele of SKP1 homolog
RED	Q9Z1M8	29.44	7.54	3	Protein Red
AP2M1	P84091	27.02	6.90	3	AP-2 complex subunit mu
CDC23	Q8BGZ4	26.83	4.69	3	Cell division cycle protein 23 homolog
EDC3	Q8K2D3	25.12	6.30	3	Enhancer of mRNA-decapping protein 3
DDX1	Q91VR5	22.53	5.81	3	ATP-dependent RNA helicase DDX1

Supplementary Table 2

Primer sequences

Name	Sequence
Expression primers	
Cdkn1c_F	GCGATCCAGACGCAGGAGCC
Cdkn1c_R	TCGCTGTTCTGCTGCGGAGG
Cdx2_F	AGTGAGCTGGCTGCCACACT
Cdx2_R	GCTGCTGCTGCTTCTTCTTGA
Elf5_F	CGAGAAGCTGAGCCGAGCCC
Elf5_R	CTCTTCCTGCCACCCGTGCG
Eomes_F	TCGCTGTGACGGCCTACCAA
Eomes_R	AGGGGAATCCGTGGGAGATGGA
Esrra_F	TCAAGGAGGGTGTGCGTCTG
Esrra_R	CCAGCTACTGCCAGAGGTCC
Esrrb_F	AGTACAAGCGACGGCTGG
Esrrb_R	CCTAGTAGATTCGAGACGATCTTAGTCA
Fgfr2_F	TGCAGCTAGGACGGTAGACA
Fgfr2_R	GTCCAGTACGGTGCTCTCTG
Gcm1_F	GCTCCACAGAGGAAGGCCGC
Gcm1_R	GTTGGTGACCGGGAAGCCGC
Gm52-(Syna)_F	CCTCACCTCCCAGGCCCCTC
Gm52-(Syna)_R	GGCAGGGAGTTTGCCCACGA
Prl2c-(Plf)_F	AACGCAGTCCGGAACGGGG
Prl2c-(Plf)_R	TGTCTAGGCAGCTGATCATGCCA
Prl3b1-(Pl2)_F	GCACTCGGGGAACAGCAGCC
Prl3b1-(Pl2)_R	ACTGCCAGCAACAGGAGTGCC
Prl3d1/2/3-(Pl1)_F	TTATCTTGGCCGCAGATGTGT

Prl3d1/2/3-(Pl1)_R	GGAGTATGGATGGAAGCAGTATGAC
Sox2_Fw	GAGTGGAAACTTTTGTCCGAGA
Sox2_Rv	GAAGCGTGTACTTATCCTTCTTCAT
Tfcp2l1_F	GAGTGCCTTTTCGGGTGCAG
Tfcp2l1_R	GCTCCCTTGGGCTTGAACAC
Tcfap2c_F	GCCGGACGCCATGTTGTGGA
Tcfap2c_R	ACCCCGGTGTGCGAGAGAGG
Nr0b1_F	TGCTCTTTAACCCAGACCTGC
Nr0b1_R	GCACTGTTCAGTTCAGCGG
Bmp4_F	AAGAGCAGAGCCAGGGAACC
Bmp4_R	GGATGTTCTCCAGATGTTCTTCGTG
Zic3_F	ACACTGGCGAGAAACCCTTC
Zic3_R	ACCGTCTGTCACAGCCTTC
Mbd3_F	AGAAGTGCCCAGGAGGTCGGG
Mbd3_R	GATCCGCCCAGGTAACGTGCC
Ovol2_F	AACTCCAGAGCTTCACGACG
Ovol2_R	GCATGTGCCGGTGGTAAACT
Ints9_F	GATCGGCAGGCTTCTCATGG
Ints9_R	TTGAGGGGAGAAGGCAGCAG
Ints4_F	GCAGCTATAAAAGCCATGTTGC
Ints4_R	GCACCTGCTCGTAGTCATCA
Ubp1_F	GGCAAAGCGAGGCAGTTGTT
Ubp1_R	TGCTCGGTTGTGAGGAGGTG
ChIP primers	
Elf5_Ch_4F	GACAAATTGGGCAAGGCTGG
Elf5_Ch_4R	AGCTCACATACCACGCAGAC
Esrrb_Ch_6F	GTGGTCCCGTGGTTTCGCAATTCT

Esrrb_Ch_6R	TCCGGTCTCAAATTCCTGCTGGGT
Eomes_Ch_9F	TACTGGGTGGAGGGTTGTGC
Eomes_Ch_9R	GCAGCTTTCCCTTTGGACCC
Tcfap2c_Ch_1F	GGAAGCCTCCTCATCAGCAA
Tcfap2c_Ch_1R	GAGGACTCCGGCCAGACTAT
Sox2_Ch_10F	GAGTTGCAGTCCCTCCGCT
Sox2_Ch_10R	ACCAAACCAAGCACAGCCC
Cdx2_Ch_5F	AAAGCAGCCAGGTCTCTTCG
Cdx2_Ch_5R	ACCACAGGCTGCTCTTTCAG
Bpm4_Ch_2F	TGCTGCTTGGATTGGAGGCT
Bpm4_Ch_2R	TGCCTTCAGGGTTGGGAGTG

Supplementary Table 3

shRNA oligonucleotides

**Name**	**Sequence**	
Esrrb_KD-1	GAGAATGGACCAGTGACA	
Esrrb_KD-2	GATTCGATGTACATTGAGA	Loh et al., 2006[4]
Esrrb_KD-3	GATCGTCTCGAATCTACTA	Loh et al., 2006[4]
Scr-1	GACGTTAGCAATCGAGCTC	Percharde et al., 2012[5]
Scr-2	GAAGGCAGTTTATTCAGTA	Loh et al., 2006[4]
Cdx2_KD-1	GTATGTCTGTGTTGTAAAT	Meissner et al., 2006[6]
Cdx2_KD-2	GCCAGAGGCAGCTAAGATA	

Supplementary Table 4

Primers used for luciferase assays

EoLF1_F	GAAACTCGGACCAGGACCTC
EoLF1_F	CTACTCGGGTTTGAGCCTCC

E5LF2_F	GCCTGTGAACAGACATTCCG
E5LF2_R	CTGGCCACCTCATAGGGAAG
E5Mut_1F	GCTCTTTTTTTACTGGTGAGAGATTAGATTATAGGTACCTTTGGAGTGAGTCAGGG
E5Mut_1R	CCCTGACTCACTCCAAAGGTACCTATAATCTAATCTCTCACCAGTAAAAAAAGAGC
E5Mut_2F	CGAGCCGAGATCCTCAGACTTATTTTGGGGATGTGACCAAATATCTACCCAA
E5Mut_2R	TTGGGTAGATATTTGGTCACATCCCCAAAATAAGTCTGAGGATCTCGGCTCG
EoMut_1F	AGGTTTTCTATGCCTCGGTGCAACATTCAAGAATTACTGGGTGGAG
EoMut_1R	CTCCACCCAGTAATTCTTGAATGTTGCACCGAGGCATAGAAAACCT
EoMut_2F	CCGGAACACCGGTCAAGATGGTCATCAACTGACCAGCAGCTTTC
EoMut_2R	GAAAGCTGCTGGTCAGTTGATGACCATCTTGACCGGTGTTCCGG

Supplementary Table 5

Antibodies

Antibody	Catalogue reference
mouse anti-Cdx2	Biogenex MU392A-UC
goat anti-Elf5	Santa Cruz sc-9645
rabbit anti-Eomes	Abcam ab23345
mouse anti-Erk1	BD 610031
mouse anti-phospho Erk1/2	Cell Signal. 9106
mouse anti-Esrrb	Perseus Proteomics PP-H6705-00
mouse anti-Flag	Sigma F1804
rabbit anti-Int1	Bethyl Laboratories A300-361A
rabbit anti-Ints9	Bethyl Laboratories A300-412A
rat anti-tubulin	Abcam ab6160
rabbit anti-Lsd1	Abcam ab17721

rabbit anti-Nr0b1	Santa Cruz sc-841X
goat anti-Sox2	R&D Systems AF2018
goat anti-AP2gamma (Tfap2c)	R&D Systems AF5059
Mouse anti-Oct4	Santa Cruz sc-5279

Supplementary References

1. Chuong, E. B., Rumi, M. A., Soares, M. J. & Baker, J. C. Endogenous retroviruses function as species-specific enhancer elements in the placenta. *Nature Genetics* **45**, 325-329 (2013).
2. Kim, J., Chu, J., Shen, X., Wang, J. & Orkin, S. H. An extended transcriptional network for pluripotency of embryonic stem cells. *Cell* **132**, 1049-1061 (2008).
3. Chen, X. *et al.* Integration of external signaling pathways with the core transcriptional network in embryonic stem cells. *Cell* **133**, 1106-1117 (2008).
4. Loh, K. M. & Lim, B. A Precarious Balance: Pluripotency Factors as Lineage Specifiers. *Cell Stem Cell* **8**, 363-369 (2011).
5. Percharde, M. *et al.* Ncoa3 functions as an essential Esrrb coactivator to sustain embryonic stem cell self-renewal and reprogramming. *Genes & Development* **26**, 2286-2298 (2012).
6. Meissner, A. & Jaenisch, R. Generation of nuclear transfer-derived pluripotent ES cells from cloned Cdx2-deficient blastocysts. *Nature* **439**, 212-215 (2006).
